# Inhibition of the PP2A activity by the histone chaperone ANP32B is long-range allosterically regulated by respiratory cytochrome *c*

**DOI:** 10.1016/j.redox.2021.101967

**Published:** 2021-04-18

**Authors:** Francisco Rivero-Rodríguez, Antonio Díaz-Quintana, Alejandro Velázquez-Cruz, Katiuska González-Arzola, Maria P. Gavilan, Adrián Velázquez-Campoy, Rosa M. Ríos, Miguel A. De la Rosa, Irene Díaz-Moreno

**Affiliations:** a Institute for Chemical Research (IIQ), Scientific Research Centre “Isla de La Cartuja” (cicCartuja), University of Seville, CSIC, Avda. Américo Vespucio 49, Seville, 41092, Spain; b Centro Andaluz de Biología Molecular y Medicina Regenerativa CABIMER, University of Seville, CSIC, University Pablo de Olavide, Avda. Américo Vespucio 24, Seville, 41092, Spain; c Institute for Biocomputation and Physics of Complex Systems (BIFI), Joint Units IQFR-CSICBIFI,and GBsC-CSIC-BIFI, Universidad de Zaragoza, 50018, Zaragoza, Spain; d Departamento de Bioquímica y Biología Molecular y Celular, Universidad de Zaragoza, 50009, Zaragoza, Spain; e Instituto de Investigación Sanitaria de Aragón (IIS Aragon), Zaragoza, Spain; f Centro de Investigación Biomédica en Red en el Área Temática de Enfermedades Hepáticas y Digestivas (CIBERehd), 28029, Madrid, Spain; g Fundación ARAID, Gobierno de Aragón, 50018, Zaragoza, Spain

**Keywords:** Cytochrome *c*, Histone chaperone, Nuclear magnetic resonance, Molecular dynamics, Protein-protein interactions

## Abstract

Repair of injured DNA relies on nucleosome dismantling by histone chaperones and de-phosphorylation events carried out by Protein Phosphatase 2A (PP2A). Typical histone chaperones are the Acidic leucine-rich Nuclear Phosphoprotein 32 family (ANP32) members, e.g. ANP32A, which is also a well-known PP2A inhibitor (a.k.a. I_1_PP2A). Here we report the novel interaction between the endogenous family member B—so-called ANP32B—and endogenous cytochrome *c* in cells undergoing camptothecin-induced DNA damage. Soon after DNA lesions but prior to caspase cascade activation, the hemeprotein translocates to the nucleus to target the Low Complexity Acidic Region (LCAR) of ANP32B; in a similar way, our group recently reported that the hemeprotein targets the acidic domain of SET/Template Activating Factor-Iβ (SET/TAF-Iβ), which is another histone chaperone and PP2A inhibitor (a.k.a. I_2_PP2A). The nucleosome assembly activity of ANP32B is indeed unaffected by cytochrome *c* binding. Like ANP32A, ANP32B inhibits PP2A activity and is thus herein referred to as I_3_PP2A. Our data demonstrates that ANP32B-dependent inhibition of PP2A is regulated by respiratory cytochrome *c*, which induces long-distance allosteric changes in the structured N-terminal domain of ANP32B upon binding to the C-terminal LCAR. In agreement with the reported role of PP2A in the DNA damage response, we propose a model wherein cytochrome *c* is translocated from the mitochondria into the nucleus upon DNA damage to modulate PP2A activity via its interaction with ANP32B.

## Highlights

• Respiratory cytochrome *c* interacts with ANP32B under DNA damage in the nucleus.
• Cytochrome *c* binding to ANP32B LCAR restores ANP32B-mediated PP2A inhibition.
• Cytochrome *c* emerges as a DNA Damage Response regulator.

## Abbreviations

ANP32: Acidic leucine-rich Nuclear Phosphoprotein 32 family
ANP32A: Acidic leucine-rich Nuclear Phosphoprotein 32 family member A
ANP32B: Acidic leucine-rich Nuclear Phosphoprotein 32 family member B
ANP32E: Acidic leucine-rich Nuclear Phosphoprotein 32 family member E
ATP: Adenosine TriPhosphate
BMRB: Biological Magnetic Resonance Bank
BSA: Bovine Serum Albumin
C*c*: Cytochrome *c*
CPT: CamPtoThecin
CRM1: ChRomosomal Maintenance 1
DAPI: 4′-6′-DiAmidino-2-PhenylIndole dihydrochloride
DDR: DNA Damage Response
DMEM: Dulbecco's Modified Eagle's Medium
DTT: DiThioThreitol
EDTA: EthyleneDiamineTetraAcetic
EGTA: Egtazic acid
FBS: Fetal Bovine Serum
GFP: Green Fluorescence Protein
HEK293T: Human Embryonic Kidney 293T
hnRNP C1/C2: heterogeneous nuclear RiboNucleoParticle C1 and C2
HSQC: Heteronuclear Single Quantum Correlation
I_1_PP2A: Inhibitor 1 of Protein Phosphatase 2A
I_2_PP2A: Inhibitor 2 of Protein Phosphatase 2A
I_3_PP2A: Inhibitor 3 of Protein Phosphatase 2A
IDRs: Intrinsically Disordered Regions
IPTG: IsoPropyl-β-D-1-ThioGalactopyranoside
ITC: Isothermal Titration Calorimetry
*K*_D_: Equilibrium dissociation constant
KLF5: Krüpper-Like transcription Factor 5
KO: KnockOut
LB: Luria-Bertani
LCAR: Low Complexity Acidic Region
LRR: Leucine-Rich Regions
Lw: Line-width
MEF: Mouse Embryonic Fibroblast
MBP: Maltose Binding Protein
MD: Molecular Dynamics
MNase: Micrococcal Nuclease
NCL: NuCleoliN
NLS: Nuclear Localization Signal
NMR: Nuclear Magnetic Resonance
NRP1: NAP-1 Related Protein 1
OPC: OPtimal 3-Charge, 4-point rigid water
PBS: Phosphate Buffer Saline
PCA: Principal Component Analysis
PFA: ParaFormAldehyde
PLA: Proximity Ligation Assay
PME: Particle Mesh Ewald
PMSF: PhenylMethylSulfonyl Fluoride
PP2A: Protein Phosphatase 2A
PTMs: Post-Translational Modifications
PVDF: PolyVinyliDene Fluoride
RPA: Replication Protein A
RT: Room Temperature
SDS-PAGE: Sodium Dodecyl Sulphate-PolyAcrylamide Gel Electrophoresis
SET/TAF-Iβ: SET/Template Activating Factor-Iβ
WT: Wild Type
Δδ_AVG_: Averaged Chemical-Shift Perturbation

## 1 Introduction

Living beings are constantly exposed to endogenous and exogenous agents that can alter the integrity of their genetic material. These agents cause lesions in DNA that can hinder essential processes such as DNA replication and transcription. If these lesions are not mitigated or properly repaired, the genetic material can suffer mutations or genomic aberrations, which threaten cell viability and can lead to cancer or genetic diseases [1].

Proper repair of these lesions is crucial for cell survival. Therefore, cells have developed specific mechanisms —collectively known as the DNA Damage Response (DDR)— which identify damage and subsequently repair it [[2], [3], [4]]. The DDR combines several signaling pathways based on alternative phosphorylation reactions, which exponentially amplify the DDR signal to achieve effective repair [5].

During DDR events, chromatin dynamics enables the entry of DNA repair factors into the damaged site. Phosphorylation of the histone H2AX at Ser139 (γH2AX) is one of the earliest events in the DDR. γH2AX signals and recruits DNA repair factors into foci to trigger post-translational modifications (PTMs) of different histones via the recruitment of histone modifiers [6,7]. These PTMs increase the mobility of histones and their eviction rates from nucleosomes. After signaling, γH2AX foci must be removed to allow the machinery to proceed with the repair process. Thus, γH2AX dephosphorylation is required for repair machinery activation [8]. This is achieved by Protein Phosphatase 2A (PP2A) which is responsible for the dephosphorylation of a range of proteins involved in the DDR, including the histone variant γH2AX [8]. Interestingly, PP2A stands out among the different cellular phosphatases, as it is ubiquitously expressed and contributes to almost 1% of the total protein content in mammalian cells [9].

Chromatin remodeling in the DDR involves histone chaperones playing a dual role: i) assisting histone eviction from nucleosomes to facilitate DNA repair mechanisms; and ii) restoring nucleosomes with newly synthesized histones when the damage is repaired [10]. In this regard, members of the Acidic leucine-rich Nuclear Phosphoprotein 32 (ANP32) family of histone chaperones have been reported to participate in transient chromatin assembly [11]. Specifically, the family member B (ANP32B) facilitates nucleosome rearrangement around the promoters of specific genes with the aid of Krüpper-Like transcription Factor 5 (KLF5) [12,13].

**Fig. 1:**
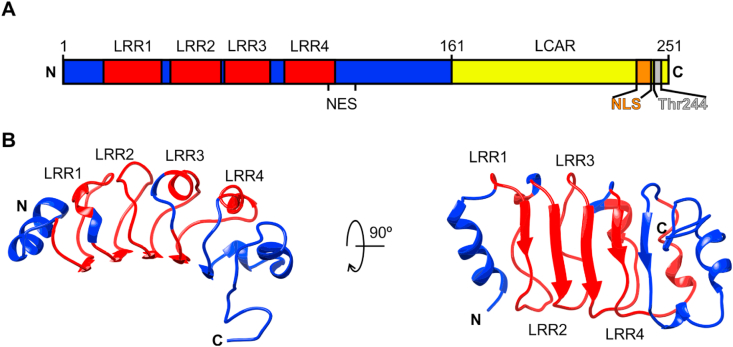
**Domain architecture and N-end structure of ANP32B.** (**A**) Schematic representation of ANP32B domain organization. The N-end structured domain of ANP32B is colored in blue, whereas the histone chaperone LCAR is represented in yellow. The four LRRs are colored in red. ANP32B Nuclear Localization Signal (NLS) is represented in orange and the histone chaperone Nuclear Export Signal (NES) is marked on the image. ANP32B Thr244 residue is represented in grey. (**B**) Ribbon representation of ANP32B (PDB: 2ELL [12]) N-terminal domain following the color scheme described in **A**. ANP32B structure is rotated 90° around the horizontal axes in each view. (For interpretation of the references to color in this figure legend, the reader is referred to the Web version of this article.)

The human ANP32 family consists of eight members, ranging from A to H, three of them (i.e. ANP32A, ANP32B and ANP32E) being conserved in vertebrates [14]. Common structural features in ANP32 proteins include a structured N-terminal domain with four Leucine-Rich Regions (LRRs) and a C-terminal Low Complexity Acidic Region (LCAR). The latter is composed of negatively charged residues lacking a discernible sequence pattern (Fig. 1A) [14]. Length (up to 100-residue) is the most distinctive feature of ANP32 LCARs when compared to other LCAR containing proteins [11]. The ANP32 LRRs form a concave groove responsible for histone binding [12], with the convex side able to interact with several ANP32 targets, such as the nuclear export protein ChRomosomal Maintenance 1 (CRM1) and PP2A [15,16] (Fig. 1B).

ANP32A and ANP32B are notable for their similarity in function and sequence [17]. The ANP32A LRR exhibits 81% sequence identity and 92% sequence homology with the LRR of ANP32B [11,18]. Recent studies indicate that ANP32A and ANP32B are essential in the viral cycle (e.g. influenza and HIV). Particularly, in host specificity, cytoplasmic transport and mRNA expression [[19], [20], [21], [22], [23]].

In proteomic studies, we found that ANP32B interacts with respiratory cytochrome *c* (C*c*) in human cells after DNA damage-induced release of the hemeprotein from the mitochondria [24]. These studies also showed that C*c* can interact with other histone chaperones, namely SET/TAF-Iβ (SET/Template Activating Factor-Iβ), NCL (NuCleoliN) and hnRNP C1/C2 (heterogeneous nuclear RiboNucleoParticle C1 and C2) in mammals [24,25], and NRP1 (NAP-1 Related Protein 1) in plants [26].

Additional data from our group unequivocally demonstrates that endogenous C*c* translocates to the nucleus soon after DNA lesions but prior to caspase activation [27]. This finding has been further corroborated by other authors using cryo-scanning transmission electron microscopy, immunostaining and subcellular fractionation [[28], [29], [30], [31]]. Shoshan-Barmatz and coworkers [29], as well as Nur-E-Kamal et al. [28] propose that nuclear C*c* regulates diverse cellular functions, including chromatin remodeling. Within this context, our data proves that nuclear C*c* hinders the binding of human SET/TAF-Iβ [27]—and its plant orthologue NRP1 [32]—to core histones, so regulating their role in chromatin reshaping. C*c* can likewise complex to 14-3-3ε, a member of the 14-3-3 protein family, to block cell survival pathways and lead cells to apoptosis [33]. However, the biological significance of C*c* binding to ANP32B during DNA damage remains unknown. A step forward in understanding how DSB-dependent DDR mechanisms could be modulated by C*c* came from our evidence of direct contacts between the hemeprotein and the LCARs of the abovementioned human SET/TAF-Iβ (and plant NRP1), thereby taking part of the extensive and branched feedback network involved in chromatin remodeling and PP2A-mediated dephosphorylation during DDR. Such a network might thus involve other LCAR-containing histone chaperones, e.g. ANPs proteins, which have also been identified as targets of C*c* in the nucleus by proteomic analyses [[24], [25], [26]].

In addition, PP2A activity can be downregulated and inhibited by histone chaperones, such as ANP32A and SET/TAF-Iβ (a.k.a. I_1_PP2A and I_2_PP2A, respectively) [16,34]. While ANP32B is a well-known histone chaperone involved in nucleosome dis/assembly, our data demonstrates that it can also act as a novel PP2A inhibitor. In this work, we determine the molecular mechanism of DNA damage-induced cellular recognition and interaction between endogenous C*c* and ANP32B in the nucleus, thereby explaining how C*c* impairs PP2A inhibition by ANP32B and competes with histones for binding with ANP32B. Our *in vitro* enzymatic assays and *in cell* γH2AX phosphorylation experiments show that PP2A inhibition relies on the ANP32B N-terminal structured LRR domain, whose inhibitory activity is finely regulated by long-distance allosteric conformational changes induced by C*c* binding to the ANP32B LCAR as part of the abovementioned LCAR-containing chaperone network. As PP2A is known to dephosphorylate several proteins involved in the DDR [8,[35], [36], [37]], we propose a molecular model in which C*c* acts as a modulator of the DNA damage response by evicting PP2A from its newly described inhibitor, ANP32B.

## 2 Material and methods

### 2.1 Cell cultures

HeLa and HEK293T (Human Embryonic Kidney 293T) cells were cultured in Dulbecco's Modified Eagle's medium (DMEM; *Sigma Aldrich*) supplemented with 10% heat-inactivated Fetal Bovine Serum (FBS, *Sigma* *Aldrich*), 2 mM l-glutamine, 100 U/mL penicillin, 100 μg/mL streptomycin, and maintained at 37 °C in a humidified atmosphere supplemented with 5% CO_2_. MEF (Mouse Embryonaric Fibroblast) WT cells were cultured in DMEM medium with 4500 mg/L glucose (*Sigma Aldrich*), supplemented with 10% heat-inactivated FBS, 2 mM l-glutamine, 100 mg/mL streptomycin, 100 U/mL penicillin and 0.11 mg/mL sodium pyruvate (*Sigma Aldrich)*. MEF C*c*^-/-^ knockout (MEF KO) cells were cultured within the same medium as MEF WT cells, but in the presence of 0.05 mg/mL uridine.

### 2.2 Immunofluorescence assays

HeLa cells (40,000 per well) were grown for 24 h, whereas MEF cells (7500 per well) were grown for 48 h; all of them were on 20 mm glass coverslips, in 24-well plates containing 500 μL of medium and treated with 20 μM CamPtoThecin (CPT) for 6 h.

In C*c* subcellular localization assays, HeLa cells were washed once in Phosphate Buffer Saline (PBS) and fixed for 20 min at Room Temperature (RT) in 2% paraformaldehyde (PFA, *Sigma Aldrich*) prepared in PBS. Then, cells were washed three times in PBS for 5 min and permeabilized for 10 min at RT with 0.5% Triton X-100 prepared in PBS. Afterwards, cells were washed three times in PBS for 5 min and incubated with blocking buffer (3% bovine serum albumin or BSA, *Sigma Aldrich*, in PBS) for 30 min at RT. Coverslips were then incubated with rabbit anti-human C*c* serum (1/200 in blocking buffer) for 1 h at RT. Coverslips were washed three times with PBS for 5 min and probed with the secondary anti-rabbit IgG–FITC antibody (1/160 in blocking buffer, *Sigma Aldrich*, catalog number F9887) for 1 h at RT, followed by three washes in PBS for 5 min. MEF cells were fixed in a solution containing ethanol and acetic acid. Cells were washed once in PBS and fixed for 20 min at −20 °C in a precooled solution of 95% (v/v) ethanol and 5% (v/v) acetic acid. Afterwards, cells were washed twice in PBS and treated with blocking buffer (10% FBS in PBS) for 30 min at RT and incubated with rabbit anti-human C*c* serum (1/200 in blocking buffer) for 1 h. Coverslips were washed three times with PBS for 5 min and probed with the secondary anti-rabbit IgG–FITC antibody (1/160 in blocking buffer, *Sigma Aldrich*, catalog number F9887) for 1 h at RT, followed by three washes in PBS for 5 min. Nuclei were stained by incubation with Hoechst dye (*Sigma Aldrich*; 200 μg/mL) for 10 min after the secondary antibody treatment, and cells were washed once with PBS for 5 min. The slides were immersed in ethanol for 2 min, dried for a few minutes, mounted using n-propyl gallate (*Sigma Aldrich*) and sealed with nail polish. HeLa cells were viewed using a Leica TCS SP5 laser-scanning microscope (*Leica Microsystems*) equipped with a 63× oil objective, whereas MEF cells were viewed using a Zeiss LSM 7 DUO scanning confocal microscope equipped with a plan-apochromat 63x/1.40 oil objective, as well as with 405 nm diode and 488 nm argon lasers for viewing Hoechst and FITC, respectively.

For C*c* and ANP32B colocalization assays, HeLa cells were washed in PBS and fixed for 20 min at RT in a 2% PFA solution freshly prepared in PBS. Afterwards, cells were washed 3 times in PBS (5 min) and permeabilized with a 0.5% Triton X-100 solution in PBS for 10 min at RT. Cells were again washed 3 times in PBS for 5 min and incubated with blocking buffer (3% BSA prepared in PBS) for 30 min at RT. The coverslips were incubated with goat anti-ANP32B (1/200 dilution, prepared in blocking buffer, *Abcam* catalog number 4224) for 1 h at RT. After three washing steps with PBS (5 min), cells were probed with donkey anti-goat-IgG-CF568 (1/200 dilution, prepared in blocking buffer, *Sigma Aldrich* catalog number SAB4600074) for 1 h at RT. Cells were then washed three times with PBS (5 min each) and incubated with rabbit anti-C*c* serum (1/200 dilution prepared in blocking buffer) overnight at 4 °C. Afterwards, cells were washed three times in PBS for 5 min and probed with goat anti-rabbit-FITC (1/160 dilution, prepared in blocking buffer, *Sigma Aldrich* catalog number F9887) for 1 h at RT, followed by three washing steps with PBS for 5 min. Nuclei were stained with Hoechst dye (*Sigma Aldrich*; 200 μg/mL) for 10 min after the secondary antibody treatment, and cells were washed once with PBS for 5 min. The slides were immersed in ethanol for 2 min, dried for a few minutes, mounted using n-propyl gallate (*Sigma Aldrich*) and sealed with nail polish. Cells were observed using a Leica TCS SP5 laser-scanning (*Leica Microsystems*) equipped with a 63× oil objective. For detecting Hoechst, FITC and Alexa Fluor568 labelling, we used a 405 nm, a 488 nm and a 543 nm laser line, respectively.

### 2.3 *In situ* Proximity Ligation Assay

HeLa cells were plated onto 12-mm-diameter coverslips, fixed with PAF, permeabilized and stained as those prepared for immunofluorescence assays. The *in situ* Proximity Ligation Assay (PLA) was performed using the Duolink™ In Situ Detection Reagent Red (*Sigma Aldrich*), anti-goat plus and anti-rabbit minus probes (*Sigma Aldrich*) following the manufacturer's instructions. Duolink In Situ Mounting Medium with DAPI was used for nuclear staining. Nuclear PLA spots were quantified using FIJI by ImageJ, in more than 90 cells per condition according to the protocol described by Prado-Martins and coworkers in 2018 [38]. Statistical analysis was carried out by ANOVA followed by Tukey's multiple comparisons test using GraphPad Prism 8.0.

### 2.4 Transfection assays

In transfection assays, 2.5 × 10^6^ HEK293T cells were cultured in 150 mm plates in complete media. After three days of growth, the old medium was removed by aspiration and fresh medium was added prior to the transfection protocol. Then, HEK293T cells were transfected with pCDNA 3.1 plasmids containing different ANP32B constructs by using calcium phosphate, as previously described [39]. After 48 h, cells were harvested from the plate surface using a scraper and washed with 5 mL of cold PBS solution to remove traces of media. After centrifuging the samples for 5 min at 400×*g*, the supernatant was removed, and cells were washed with 5 mL of cold PBS solution. Then, the supernatant was discarded by centrifugation and cells were resuspended in a specific lysis buffer for each assay.

### 2.5 Pull-down assays

Transfected HEK293T cells were resuspended in a lysis buffer containing 10 mM Tricine-NaOH pH 8.5, 1 mM PhenylMethylSulfonyl Fluoride (PMSF) and cOmplete protease inhibitors (*Roche*) to perform C*c* pull-downs; or 25 mM Tris-HCl pH 8.0, 50 mM NaCl, 20 mM imidazole, 5% glycerol, 1% NP-40, 1 mM PMSF and cOmplete protease inhibitors for the PP2A pull-downs. Cells were then lysed by sonication (10 s, 10% amplitude, on ice). Afterwards, cell debris was discarded by 15 min centrifugation at 16,300×*g* at 4 °C.

For C*c* pull-down assays, cell extracts were incubated with 100 μg of recombinant C*c* for 16 h at 4 °C in batch. Afterwards, cell extracts were incubated with a carboxymethyl cellulose matrix (*Whatman*), previously equilibrated with lysis buffer for 30 min at 4 °C in batch. To remove any non-specific binding, the matrix was washed three times with 1 mL of washing buffer containing 10 mM Tricine-NaOH pH 8.5, 20 mM NaCl, 1 mM PMSF and cOmplete protease inhibitors. Then, the C*c*:ANP32B complex was released from the matrix with an elution buffer containing 10 mM Tricine-NaOH pH 8.5, 360 mM NaCl, 1 mM PMSF and cOmplete protease inhibitors. Results were assessed by Western-Blot using antibodies against *c*-myc clone 4A6 (*EMD Millipore*, catalog number #05-724) and C*c* (obtained by immunizing male rabbits with recombinant C*c*).

For PP2A pull-down assays, endogenous PP2A was snared from cell extracts by profiting on its affinity towards divalent cations [40,41], using a Ni-NTA matrix (*Generon*) for 16 h at 4 °C in batch. Afterwards, the matrix was washed three times with lysis buffer. The PP2A:ANP32B complex was eluted by adding a buffer containing 25 mM Tris-HCl pH 8.0, 50 mM NaCl, 500 mM imidazole, 1 mM PMSF and cOmplete protease inhibitors. Results were assessed by Western-Blot, using antibodies against *c*-myc and the catalytic subunit of PP2A clone 1D6 (*EMD Millipore*, catalog number #05-421).

As a control, extracts from HEK293T cells transfected with an empty vector were processed in the same way as the experimental samples to discard the possibility of non-specific binding.

### 2.6 Western-Blot assays

Proteins were separated by Sodium Dodecyl Sulphate-PolyAcrylamide Gel Electrophoresis (SDS-PAGE) (15% acrylamide) and transferred onto PolyVinyliDene Fluoride (PVDF) membranes (*EMD Millipore*) using a Mini Trans-Blot electrophoretic transfer cell (*Bio-Rad*). Membranes were blocked with 5% fat-free dry milk in PBS-0.1% Tween 20. Immunoblotting was performed with specific primary antibodies against targeted proteins. HRP-conjugated secondary antibodies were used for detection depending on the primary antibody host (Goat IgG, *Dako,* ref. P0449; Mouse IgG, *Sigma Aldrich,* ref. A9044; Rabbit IgG, *Sigma Aldrich,* ref. A0545). The immunoreactive bands were detected using Amersham ECL Plus Western Blotting Detection Reagents (*GE Healthcare Life Sciences*).

### 2.7 Caspase-3 activation assays

For caspase-3 activation assays, 200,000 HeLa cells were cultured in 35 mm wells using 2 mL of complete DMEM. After 24 h of growth, cells were treated with 20 μM CPT for 4, 6, 12, 24 and 48 h. Afterwards, cells were harvested with a scrapper, and the wells were washed with 2 mL of PBS. Then, cells were centrifuged at 3,000×*g* for 5 min and the supernatant was discarded. Cells were washed with 2 mL of PBS to remove media traces and further centrifuged at 3,000×*g* for 5 min. Cell pellets were resuspended in 200 μL of TR3 buffer (containing 10 mM Na_2_HPO_4_, 10% glycerol and 3% SDS) and mixed with a vortex to homogenize the mixture. Following this, cells were disrupted by sonication (3 s, 30% amplitude) and cell debris were discarded upon centrifugation (10 min, 16,000×*g*). Afterwards, protein concentration was determined by using the DC Protein Assay Kit (*BioRad*) following the manufacturer's instructions. Samples for caspase-3 cleavage detection were homogenized to 40 μg of total protein per sample, whereas those used as loading control were at 13.5 μg. Then, samples were heated at 100 °C for 5 min in presence of protein loading buffer and loaded into a 15% SDS-PAGE. Procaspase-3, caspase-3 and GAPDH were detected by Western-Blot using a mouse Anti-Caspase-3 (*Santa Cruz Biotechnology*, sc-271028) and mouse Anti-GAPDH (*Santa Cruz Biotechnology*, sc-47724).

### 2.8 DNA constructs

The DNA encoding full-length ANP32B (ANP32B_1-251_) was obtained via polymerase chain reaction (PCR) using cDNA (Geneservice, UK) and inserted into the pET28a(+) bacterial expression vector with a N-terminal 6xHis-tag. The oligonucleotides used in the PCR were 5′-AGCCATATGATGGACATGAAGAGGAGG-3′ and 5′-GTGGTGCTCGAGTCAATCATCTTCTCC-3’. ANP32B N-end LRR domain (ANP32B_1-161_) in pET28a(+) was obtained by mutagenic PCR for pET28a(+)-ANP32B_1-251_ using 5′-GATGCCGAGTAAGATGGTGTG-3′ and 5′-CACACCATCTTACTCGGCATC-3′ as forward and reverse primers, respectively. An ANP32B construct lacking its Nuclear Localization Signal (NLS) sequence (ANP32B_1-231_) in pET28a(+) was obtained by mutagenic PCR from pET28a(+)-ANP32B_1-251_ using 5′- GATGAAGAGGAGTAAGAAGGTGGGAAA-3′ and 5′-TTTCCCACCTTCTTACTCCTCTTCATC-3′ as forward and reverse primers, respectively.

ANP32B_1-251_ and ANP32B_1-161_ constructs were cloned into the mammalian expression vector pcDNA 3.1—kindly gifted by Prof. Khalid Iqbal, *New York State Institute for Basic Research U.S.A.*—using the In-Fusion HD Cloning kit (*Clontech*) following the manufacturer's instructions. Primers used to linearize the pcDNA 3.1 were 5′-GAGCAGAAACTCATCTCTGAA-3′ as a forward primer and 5′-GGATCCGAGCTCGGTACCAAG-3′ as a reverse primer. The ANP32B inserts were amplified using the primers 5′-ACCGAGCTCGGATCCATGGACATGAAGAGGAGGATC-3′ and 5′-GATGAGTTTCTGCTCATCATCTTCTCCTTCATCATC-3′ for ANP32B_1-251_; and 5′-ACCGAGCTCGGATCCATGGACATGAAGAGGAGGATC-3′ and 5′-GATGAGTTTCTGCTCCTCGGCATCTGAGTCAGGTGC-3′ for ANP32B_1-161_. D31A mutant of ANP32B_1-251_ in pcDNA 3.1 plasmid was obtained by mutagenic PCR from a pcDNA 3.1-ANP32B_1-251_ plasmid, using 5′-AAATCAAATGCCGGAAAAATT-3′ and 5′-AATTTTTCCGGCATTTGATTT-3′ as forward and reverse primers, respectively.

Maltose Binding Protein (MBP) was cloned into a pcDNA 3.1 plasmid using an In-Fusion HD Cloning Kit (*Clonetech*) following the manufacturer's instructions. pcDNA 3.1 was linearized using the primers described above. The MBP insert was amplified using the following primers: 5′-ACCGAGCTCGGATCCATGGCACACCATCACCACCAT-3′ forward primer and 5′-GATGAGTTTCTGCTCCGGGCCCTGAAACAGAACTTC-3′ reverse primer. The LCAR domain (ANP32B_162-251_) construct was cloned at the C-end of pcDNA 3.1 - MBP. Primers used to linearize the pcDNA 3.1- MBP were 5′-GAGCAGAAACTCATCTCTGAA-3′ forward primer and 5′-CGGGCCCTGAAACAGAACTTC-3′ reverse primer. ANP32B_162-251_ insert was amplified using the forward primer 5′-CTGTTTCAGGGCCCGGTGGATGGTGTGGATGAAGAG-3′ and the reverse primer 5′-GATGAGTTTCTGCTCATCATCTTCTCCTTCATCATC-3’.

DNA coding for C*c* was cloned into the pBTR1 vector [42] which also encoded the yeast heme lyase to guarantee proper protein folding.

### 2.9 Protein expression and purification

C*c* was expressed in *E. coli* BL21 (DE3) cells as previously described with minor modifications [[43], [44], [45], [46]]. After electroporation, clones containing the plasmid encoding C*c* were selected by growing them for 16 h in LB-agar plates supplemented with 100 μg/mL ampicillin. A single clone was selected and grown at 37 °C in 25 mL of Luria-Bertani (LB) medium. 2.5 mL of pre-culture was used to inoculate 2.5 L of LB medium and incubated at 30 °C under agitation for 24 h. Cells were then harvested by centrifugation (8,900×*g* for 10 min) and resuspended in 1.5 mM borate buffer pH 8.5. Lysis was performed by sonication for 4 min, and the soluble protein was separated from cellular debris by centrifugation (47,500×*g* for 30 min). For NMR measurements, ^15^N-labelled C*c* was produced in M9 minimal media with ^15^NH_4_Cl as a nitrogen source. C*c* purification was carried out by ionic chromatography with a carboxymethyl cellulose matrix (*Whatman)*. Fractions containing pure C*c* were dialyzed and concentrated in a 20 mL Pierce Protein Concentrator PES 3.0 M.W.C.O. (*Thermo Scientific*) until the desired protein concentration was achieved.

The expression of ANP32B_1-251_ and ANP32B_1-231_ recombinant proteins was performed in *E. coli* BL21 (DE3) pLysS cells, whereas ANP32B_1-161_ expression was carried out in BL21 (DE3) cells. Pre-cultures were inoculated with a single clone selected from a LB-agar plate supplemented with 100 μg/mL kanamycin and 50 μg/mL chloramphenicol or 100 μg/mL kanamycin from a transformation with the pET28a(+)-ANP32B_1-251_/ANP32B_1-231_ or pET28a(+)-ANP32B_1-161_ plasmid, respectively. Pre-cultures were grown at 37 °C for 16 h. Then 2 L of LB media was inoculated at a ratio of 1:1000 with the above-mentioned pre-cultures. Cultures were grown at 37 °C until the Optical Density (OD)_600nm_ reached 0.4–0.6, at which point the expression of the protein was induced by adding 1 mM IsoPropyl-β-D-1-ThioGalactopyranoside (IPTG). Afterwards, bacteria expressing ANP32B_1-251_ or ANP32B_1-231_ were grown at 16 °C for 16 h, whilst those expressing ANP32B_1-161_ were incubated at 37 °C. Cells were harvested by centrifugation (8,900×*g* for 10 min) and resuspended in a lysis buffer containing 20 mM Tris-HCl pH 8.0, 100 mM NaCl, 1 mM DiThioThreitol (DTT), 1 mM PMSF, 0.2 mg/mL lysozyme, 0.02 mg/mL DNase and cOmplete protease inhibitors (*Roche*). Cells were ruptured by sonication (cycles of 30 s at 40% of amplitude, 60 s of rest, 3 min total time, on ice). Afterwards, cellular debris was separated by centrifugation at 47,500×*g* for 30 min. The supernatant was loaded onto a Ni-NTA matrix (*Generon*), previously equilibrated with a lysis buffer, and incubated for 1 h in batch at 4 °C. Recombinant proteins were eluted using a non-continuous imidazole gradient and the purity was checked by SDS-PAGE. Fractions containing the purified ANP32B proteins were mixed and dialyzed for 24 h at 4 °C against a lysis buffer lacking imidazole. For ANP32B_1-251_ and ANP32B_1-231_ constructs, impurities were removed by an additional purification step using a Superdex 200 10/300 GL (*GE Healthcare*) and 10 mM sodium phosphate pH 7.4 as running buffer at 0.5 mL/min flow rate. Protein purity was later checked by SDS-PAGE and concentrated using a 20 mL Pierce Protein Concentrator PES 3.0 M.W.C.O. (*Thermo Scientific*).

### 2.10 Nuclear Magnetic Resonance

Nuclear Magnetic Resonance (NMR) titrations of reduced ^15^N-labelled C*c* samples with ^14^N-non-labelled ANP32B constructs were recorded and monitored at 25 °C by 1D ^1^H and 2D [^1^H–^15^N] Heteronuclear Single Quantum Correlation (HSQC) spectra in a Bruker Avance-III 600 MHz equipped with a cryoprobe. 30 μM ^15^N-labelled C*c* in 10 mM sodium phosphate buffer pH 7.4 was titrated with ^14^N ANP32B_1-251_ dialyzed in the same buffer. To ensure that C*c* remained in its reduced redox state and to adjust the lock signal of the NMR spectrometer, 0.1 M sodium ascorbate and 10% D_2_O were respectively added to the samples. Titration of reduced ^15^N-labelled C*c* with the ANP32B_1-161_ construct was performed in 5 mM sodium phosphate buffer pH 6.4. Samples were prepared in NMR tubes (*Shigemi Inc.*) to a final volume of 0.3 mL and the pH value was checked at each step of the titrations. Data was acquired and processed using TopSpin NMR 3.5pl7 software (*Bruker*). Line broadening and chemical-shift perturbation analyses were performed using the Sparky 3 NMR assignment tool (T.D. Goddard and D.G. Kneller, University of California – San Francisco, US). The NMR signal for ^1^H and ^15^N nuclei of reduced C*c* was already available (Biological Magnetic Resonance Bank [BMRB] accession number: 5406) [47]. Chemical-shift perturbations (Δδ_AVG_) in ^15^N-labelled C*c* titrations with ^14^N-ANP32B_1-251_ were calculated as previously described [48,49].

### 2.11 Isothermal Titration Calorimetry

Isothermal Titration Calorimetry (ITC) experiments involving ANP32B_1-251_, ANP32B_1-231_ and ANP32B_1-161_ were performed using a Nano ITC Low Volume instrument (*TA Instruments, U.S.A.*). The reference cell was filled with distilled water. Titration experiments of ANP32B_1-251_ or ANP32B_1-231_ with C*c* consisted of 17 successive 2.91 μL injections, from a stock of reduced C*c* (400 μM) into the 161 μL sample cell, containing 50 μM or 40 μM of ANP32B_1-251_ and ANP32B_1-231_, respectively. C*c* and ANP32B protein samples were dialyzed against 10 mM sodium phosphate buffer pH 7.4. Assays performed at moderate and high ionic strength were carried out at either 20 mM sodium phosphate pH 7.4 with 50 mM NaCl or 10 mM sodium phosphate pH 7.4 with 150 mM NaCl. Titration experiments between ANP32B_1-161_ and reduced C*c* consisted of 17 successive injections from a stock of 400 μM reduced C*c* into the sample cell containing 50 μM ANP32B_1-161_. Both C*c* and ANP32B dilutions were dialyzed in 10 mM sodium phosphate buffer pH 7.4. Homogeneous mixing was achieved by maintaining the stirring speed at 300 rpm. ANP32B_1-251_ and ANP32B_1-231_ titration data was analyzed using Origin 7.0 (OriginLab) with models based on a single- or two-ligand binding sites. Cooperativity between both sites in the case of two binding sites could not be observed. ANP32B_1-161_ titration data was processed and analyzed using NanoAnalyze software (*TA Instruments*) and Origin 7.0.

### 2.12 Binding competition assays

1D ^1^H NMR spectra were recorded in a 700 MHz Bruker-Avance III furnished with a 5 mm TXI cryoprobe to monitor the signal of Met80-ε-CH_3_ in a sample of 13 μM unlabeled reduced C*c,* either free or in the presence of 6.5 μM ANP32B_1-251_ and calf thymus histones (10–40 μg, *Sigma Aldrich*). To discard non-specific interactions, a 1D ^1^H NMR spectrum of 13 μM reduced ^14^N C*c* was acquired in the presence of either 40 μg of BSA or 40 μg of calf thymus histones. All measurements were prepared in 3 mm NMR tubes containing samples with a final volume of 0.2 mL. These samples were prepared in 10 mM sodium phosphate buffer pH 7.4, 10% D_2_O with 65 μM sodium ascorbate to maintain C*c* in its reduced redox state. Spectra were acquired and processed using TopSpin 3.5pl7 NMR software (*Bruker*) and graphed using OriginLab 2018 software.

### 2.13 Nucleosome assembly assays

The nucleosome assembly activity of ANP32B_1-251_ was tested using a micrococcal MNase digestion assay, as described by González-Arzola et al. [32]. The pBlueScript SK (−) was purified by alkaline lysis as previously described [27]. 1 μg of pBlueScript II SK (−) plasmid was relaxed with 2.5 U of Topoisomerase I from wheat germ (*Promega*) in 50 μL of TopoI buffer, containing 10 mM Tris-HCl pH 8.0, 50 mM NaCl, 3.5 mM MgCl_2_, 1 mM DTT, 2 mM Adenosine TriPhosphate (ATP) and 0.1 mg/mL BSA at 37 °C for 3 h. 4 μg of Core Histones HeLa (*EMD Millipore*) were incubated with 6 μg of WT ANP32B_1-251_ for 3 h at 37 °C in 50 μL of assembly buffer, containing 10 mM Tris-HCl pH 8.0, 50 mM NaCl, 2 mM MgCl_2_, 1 mM DTT, 2 mM ATP and 0.1 mg/mL BSA. C*c* was also incubated with the histone-chaperone mixture using the same conditions as before. TopoI-relaxed pBlueScript SK II plasmid was combined with the histone-chaperone mixture, with and without C*c*, for 3 h at 37 °C. Afterwards, 5 mM CaCl_2_ was added to the mixture before the addition of MNase to improve its activity. The sample was then incubated with 7.5 U of MNase for 30 s at 37 °C. MNase digestion was halted by adding stop buffer, containing 20 mM EthyleneDiamineTetraAcetic acid (EDTA) pH 8.0, 1% SDS and 0.2 mg/mL Proteinase K and incubated for 30 min at 37 °C. Digested DNA was extracted using FavorPrep GEL/PCR Purification Mini Kit (*Favorgen*). Purified DNA was subjected to electrophoresis in 2% agarose gel, run in Tris-borate-EDTA buffer for 1 h at 80 V and visualized by ethidium bromide staining.

### 2.14 Protein phosphatase 2A assays

Relative PP2A activity was determined as previously described [34] with minor modifications. HEK293T cells transfected with the ANP32B constructs in pcDNA 3.1 plasmids were resuspended in a lysis buffer containing 10 mM Tris-HCl pH 7.4, 50 mM NaCl, 1 mM egtazic acid (EGTA) and 1 mM EDTA. Afterwards, cells were lysed by sonication (10 s, 10% amplitude, on ice). Cell debris was removed by centrifugation for 15 min at 16,300×*g*.

120 μg of cell extract was incubated with increasing concentrations of recombinant C*c* in reaction buffer (50 mM Tris-HCl pH 7.4; 0.1 mM CaCl_2_; 2.5 mM NiCl_2_) containing 1 mg/mL *p*-nitrophenyl phosphate (*Sigma Aldrich*) as an enzyme substrate for 15 min at 37 °C. Reactions were stopped by adding 13% K_2_HPO_4_. Activity was monitored by measuring the absorbance at 410 nm in a plate reader spectrophotometer (BIO-RAD, model 680). Data was normalized using cell extracts transfected with an empty vector but the same concentrations of C*c* to discard any impact the hemeprotein may have on PP2A activity. Each experimental data point was the mean value ± SD of three independent measurements.

### 2.15 *In cell* γ-H2AX detection assays

For γH2AX dephosphorylation assays, 40,000 MEF WT and C*c*^-/-^ KO cells were grown on glass slides within 22 mm diameter wells. After 48 h of growth, cells were exposed to 10 μM CPT for 1 h to induce DNA damage. Afterwards, CPT-containing media was removed and replaced with newer pre-warmed media. Cells were allowed to recover from CPT-induced DNA damage for 0 or 3 h, and washed twice with PBS prior to fixation in 3.7% PFA in PBS (15 min at RT). In each case, cells were rinsed twice with 1% BSA solution in PBS for 5 min, and permeabilized by incubating the coverslips for 20 min at RT in a 0.5% Triton X-100 solution in PBS. After repeating the two washing steps with 1% BSA-PBS solution, cells were incubated in a 3% BSA-PBS solution to prevent non-specific binding of the primary antibody. Right after, cells were incubated with a primary antibody against γ-H2AX (*Millipore*, Cat.# 05-636, 1/250 dilution prepared in blocking buffer) for 1 h at RT. Coverslips were washed 3 times with 0.1% Tween-20 solution prepared in PBS (5 min at RT). Cells were then probed with the secondary antibody Goat Anti-Mouse IgG Alexa Fluor 568 (*Abcam*, Cat.#ab175473, 1/500 dilution in blocking buffer) for 1 h at RT. Afterwards, glass slides were washed twice with a 0.1% Tween-20 solution in PBS (5 min at RT). Cell nuclei were stained by incubating the cells in a 200 μg/mL Hoechst solution in PBS for 10 min at RT. Cells were washed once in PBS and dried by incubation in ethanol for 2 min and air dried. Finally, glass slides were mounted using Vectashield Antifade Mounting Medium (Vector Laboratories) and sealed with nail polish. Cells were observed using a Leica TCS SP5 laser-scanning (*Leica Microsystems*) equipped with a 63× oil objective. For detecting Hoechst and Alexa Fluor 568 fluorescence, 405-nm and 543-nm laser lines were respectively used. γ-H2AX signal intensity was integrated using *ImageJ* (*National Institute of Health*, United States of America).

### 2.16 Docking calculations

*Ab initio* docking calculations on the ANP32B_1-161_:PP2A complex were performed using Hex Protein Docking [50]. For this purpose, PP2A heterotrimeric holoenzyme structure (PDB: 2IAE [51]) was set up as the target protein, and the ANP32B_1-161_ structure (PDB: 2ELL [12]) as the probe. The correlation type was set up to “*shape and electro*”, using both CPU and GPU computing devices. FTT mode was set to 3D and 50,000 solutions were explored. Afterwards, the best 100 solutions were stored and analyzed.

### 2.17 Molecular dynamics

Molecular dynamics (MD) computations were carried out on the ANP32B full-length protein using the AMBER 16 package and the AMBER-2003 force field [52,53]. The LCAR of ANP32B_1-251_ was added using the built-in module MODELLER [54] of Chimera 1.11.2 [55].

Simulations of free WT ANP32B_1-251_, ANP32B_1-251_ D31A mutant, ANP32B_1-161_ construct, WT ANP32B_1-251_ with two C*c* molecules (PDB ID: 1J3S [47]) on the surface of LCAR in ANP32B and the ANP32B_1-251_:PP2A complex were carried out with periodic boundary conditions using an orthorhombic cell geometry. The minimum distance between protein and cell faces was set to 10 Å. Particle Mesh Ewald (PME) electrostatics were set with the Ewald summation cut-off at 9 Å. To compensate and neutralize the charges of the system, sodium counter-ions were added according to the total charge of the protein. Water solvation of the structures was performed using OPtimal 3-Charge, 4-point rigid water model (OPC) water molecules [56]. Solvent and counter-ions were subjected to 2000 steps of energy minimization, followed by 300 ps NPT-MD computations using isotropic molecule position scaling and a pressure relaxation time of 1 ps at 298 K. The density of the system reached a plateau during the first 150 ps. Later, the whole system was subjected to energy minimization and NVT MD computations at 298 K. SHAKE algorithms were used throughout the calculation to constrain bonds involving hydrogen atoms. The CPPTRAJ module of AMBER was later used for trajectory analysis [57].

## 3 Results

### 3.1 Respiratory mitochondrial cytochrome *c* shuttles into the nucleus where it interacts with ANP32B upon DNA damage

**Fig. 2:**
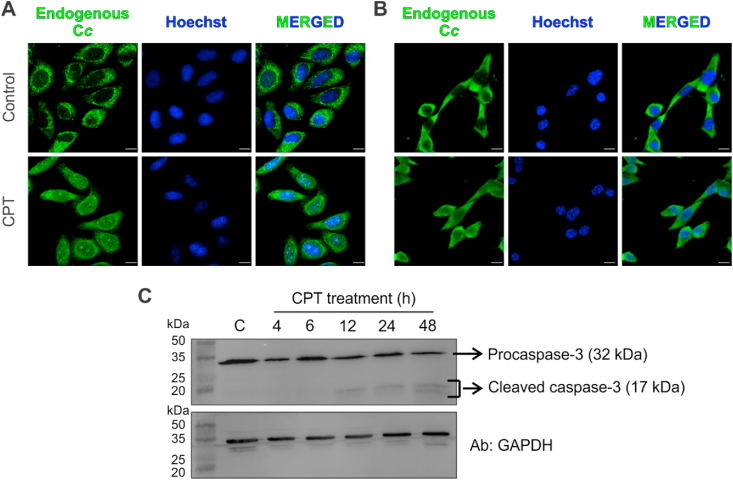
**CPT-induced nuclear translocation of endogenous cytochrome *c*.** Immunofluorescence analysis of endogenous C*c* in HeLa (**A**) and MEF (**B**) cells, upon treatment with 20 μM CPT for 6 h. Colocalization of green C*c* fluorescence and blue nuclear staining is shown in the merged images in (**A**) and (**B**). Scale bars are 10 μm. (**C**) Activation of caspase-3 in HeLa cells upon 4, 6, 12, 24 and 48 h of CPT treatment (20 μM). *Upper panel*— Western-blot against caspase-3, showing the appearance of the active form of caspase-3 (cleaved caspase-3) after 12 h of CPT-induced DNA damage. *Lower panel*— Western-blot against GAPDH as loading control. (For interpretation of the references to color in this figure legend, the reader is referred to the Web version of this article.)

Translocation of endogenous C*c* into the nucleus upon DNA damage induction by 6 h CPT treatment was detected by immunofluorescence assays. Fig. 2 shows C*c* in the nucleus in both HeLa (Fig. 2A) and MEF WT (Fig. 2B) cells upon CPT treatment, as previously described [[27], [28], [29]]. To check whether apoptosis was initiated simultaneously with nuclear translocation of C*c*, activation of caspase-3 was monitored by Western-blot analysis in HeLa cells after 4, 6, 12, 24 and 48 h of CPT treatment. Interestingly, our results show that procaspase-3 remains non-cleaved during the first 6 h after CPT treatment, becoming active only at long time after DNA damage, starting at 12 h (Fig. 2C).

**Fig. 3:**
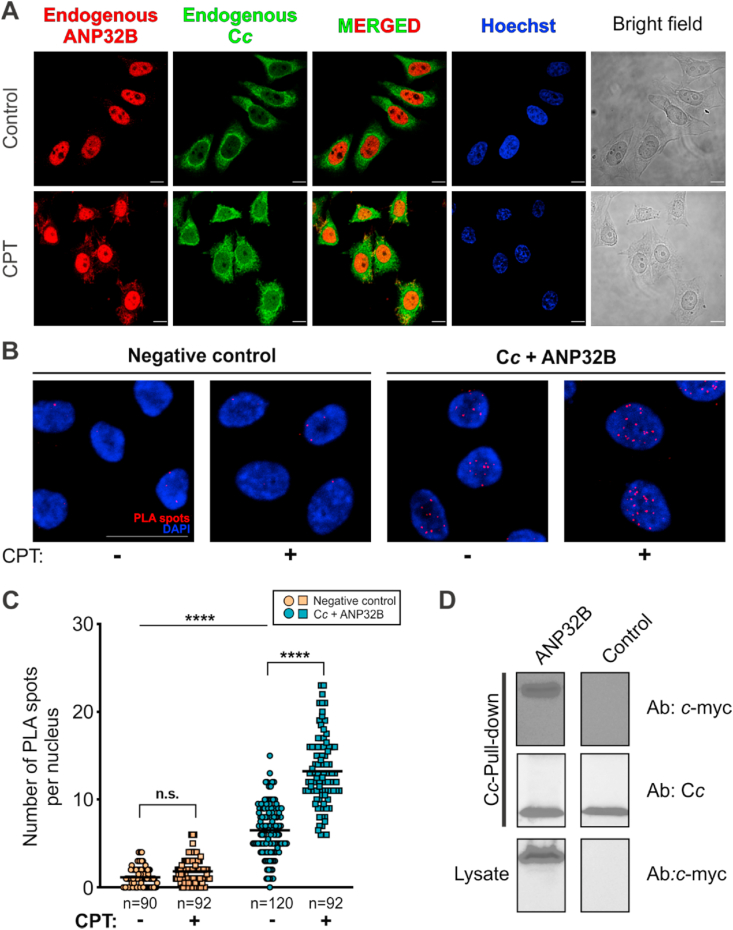
**Cytochrome *c* colocalizes and interacts with ANP32B in the cell nucleus upon DNA damage.** (**A**) Colocalization assays between endogenous C*c* and endogenous ANP32B in non-treated (control) and CPT-treated HeLa cells. Subcellular distribution of C*c* and ANP32B was visualized with an anti-C*c* antibody (green fluorescence) and an anti-ANP32B antibody (red fluorescence), respectively. Nuclei were stained with Hoechst (blue fluorescence). The merged images correspond to the overlaid images of C*c* and ANP32B. Scale bars in panel **A** are 10 μm. (**B**) *In situ* Proximity Ligation Assay (PLA) for detection of C*c*:ANP32B complexes in HeLa cells following CPT treatment for 6 h. Representative confocal maximal projections of untreated (*top*) and CPT-treated (*bottom*) cells, with PLA spots in red and nuclei in blue. As a negative control, goat ANP32B antibody was used together with an unspecific rabbit primary antibody and the two PLA probes (−/+). Scale bar represents 25 μM. (**C**) Scatter plot showing quantification of the PLA signal as the number of PLA spots per nucleus. Data were collected from three independent experiments as that shown in panel **B**. Black lines represent the mean of the data collected. ****p < 0.0001; n.s., non-significant. (one-way ANOVA followed by a Tukey's post hoc test). (**D**) *Upper panel* — Western-Blot against *c*-myc tag, encoded at the C-terminal of ANP32B, after performing the pull-down assays. *Middle panel* — Western-Blot against C*c*, demonstrating that recombinant C*c* was captured in the carboxymethyl cellulose in all cases. *Lower panel* — Western-Blot against *c*-myc tag of cell lysates as loading and transfection control. (For interpretation of the references to color in this figure legend, the reader is referred to the Web version of this article.)

We further monitored the colocalization between endogenous C*c* and endogenous ANP32B in the nucleus within the 6 h timeframe of CPT-induced DNA damage. Under such conditions, C*c* colocalizes in the nucleus with the histone chaperone in HeLa cells (Fig. 3A). To demonstrate that the physical interaction between both endogenous C*c* and ANP32B occurs, Proximity Ligand Assay (PLA) assays were performed. Under CPT-induced DNA damage, a substantial number of PLA signals confirmed that i) C*c* and ANP32B interact with each other, and ii) such an interaction occurs in the cell nucleus (Fig. 3B). A few PLA spots were likewise observed in the nucleus of untreated cells, suggesting that a small pool of C*c* molecules might localize at the nucleus under homeostasis (Fig. 3B). This agrees with other authors reporting that HeLa cells suffer basal levels of DNA damage under homeostasis [58,59]. However, quantification of the number of PLA spots in CPT-treated and untreated cells unequivocally reveals statistically significant differences (p < 0.0001) between both situations (Fig. 3C).

Pull-down assays also corroborated the physical contact between C*c* and ANP32B in cell extracts obtained from HEK293T ANP32B-*c*-myc-transfected cells. Recombinant C*c* was used as bait to catch ANP32B. The resulting C*c*:ANP32B complex was co-purified using a carboxymethyl cellulose column, and immunodetection of ANP32B-*c*-myc in the C*c* pull-down samples corroborated the *in cell* interaction between ANP32B and C*c* (Fig. 3D). ANP32B-*c*-myc was not detected in the non-transfected C*c*-pull-down samples used as a control (Fig. 3D).

### 3.2 The low complexity acidic region of ANP32B leads the binding to cytochrome *c* at the heme surroundings

**Fig. 4:**
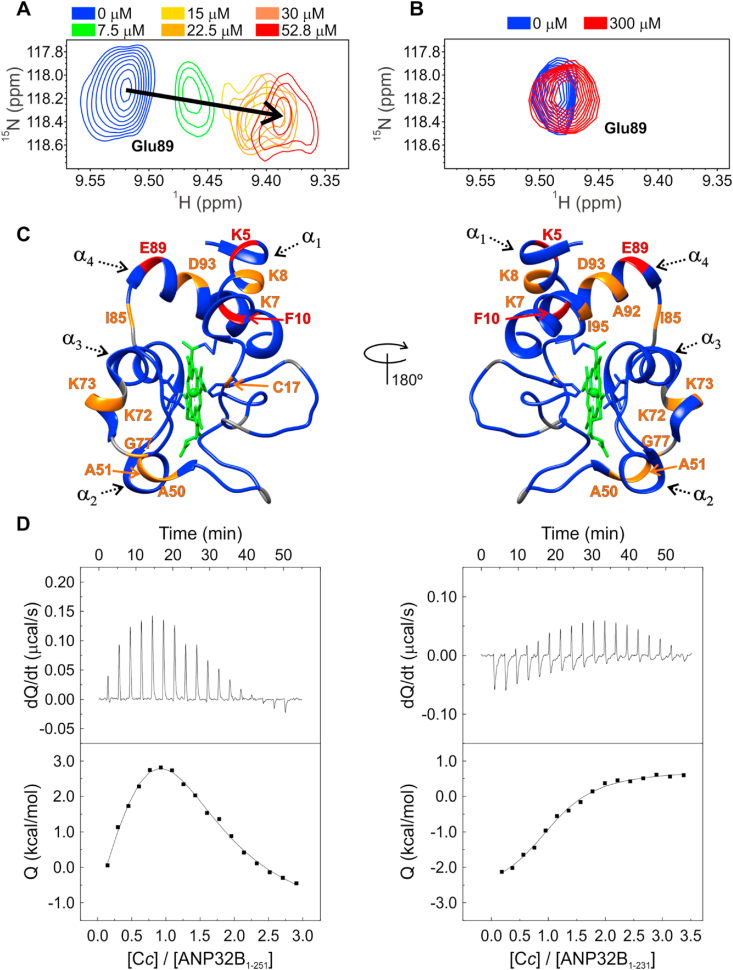
**ANP32B low complexity acidic region drives C*c*:ANP32B complex formation.** Detailed view of the superimposed [^1^H–^15^N] 2D HSQC spectra of ^15^N-labelled C*c* upon titration with increasing concentrations of ANP32B_1-251_ (**A**) and ANP32B_1-161_ (**B**). Color code for ANP32B_1-251_ concentration is shown in the panels. Displayed resonance corresponds to C*c* Glu89 amide group. (**C**) Ribbon representation of C*c* colored according to Δδ_AVG_ of the amide signals upon titration with ANP32B_1-251_. Δδ_AVG_ categories are colored as follows: small <0.050 ppm (blue), medium 0.050–0.075 ppm (orange) and large >0.075 ppm (red). Prolines and unassigned residues are in grey, and the heme group is in green. C*c* molecule is rotated 180° around vertical axes in each view. The four alpha-helices of C*c*—named from α_1_ to α_4_— are indicated by dashed arrows. C*c* PDB ID: 1J3S [47] (**D**) ITC analysis of the interaction between reduced C*c* with ANP32B_1-251_ (*left panel*) and ANP32B_1-231_ (*right panel*). Thermograms and binding isotherms are shown in the *upper* and *lower* panels, respectively. (For interpretation of the references to color in this figure legend, the reader is referred to the Web version of this article.)

Colocalization, PLA and pull-down assays corroborated the formation of the endogenous C*c*:ANP32B complex in cells with DNA damage. Understanding the molecular mechanisms driving this interaction entailed identifying the ANP32B domain—either the LRR or LCAR (see Fig. 1)—responsible for binding with the hemeprotein. Hence, we tested the ability of the two domains of ANP32B to bind C*c* by monitoring the behavior of the reduced C*c* amide signal in NMR titration assays. Specific amide signals (Fig. S1A) in the 2D [^1^H–^15^N] HSQC spectra of C*c* underwent chemical-shift perturbations (Δδ_AVG_) upon the addition of *full-length* ANP32B (ANP32B_1-251_; Fig. 4A). A residue-specific line-width (Lw) broadening pattern was also evident at a 1:1 C*c*:ANP32B_1-251_ ratio (Fig. S1B). Altogether, our data indicates that specific signals undergo an intermediate chemical exchange with a lifetime within the 10 ms range. Similarly, other histone chaperones (e.g. human SET/TAF-Iβ and plant NRP1) form transient complexes with C*c* with dissociation constant (*K*_D_) values laying within the micromolar range [27,32]. On the contrary, the C*c* amide NMR signals were insensitive to titrations with an ANP32B construct lacking the LCAR domain (ANP32B_1-161_) even at a 1:10 (C*c*:ANP32B_1-161_) ratio (Fig. 4B). Hence, C*c* binds the histone chaperone ANP32B via its LCAR region.

Mapping Δδ_AVG_ and line-broadening onto the C*c* surface (Fig. 4C and Fig. S1C, respectively) revealed the patch interacting with ANP32B_1-251_. Notably, residues undergoing the largest Δδ_AVG_ (>0.075 ppm) are placed in three discrete—and distant—structural areas of C*c*, namely α_1_, α_2_ and α_4_ helices (Fig. 4C). Other residues with Δδ_AVG_ ranging between 0.050 and 0.075 ppm are placed within the proximities of such areas (Fig. S1A), whereas the amide groups at flexible loops experience specific line broadening (Fig. S1C).

The sparse pattern of C*c* surface residues affected by ANP32B binding indicates that the ANP32B LCAR contacts to C*c* using distant regions, clamping the hemeprotein through diverse points. This finding suggests that C*c* and ANP32B engage in a fuzzy ensemble, likely following a flanking model [60]. According to this model, the unstructured protein regions bind their partners through short recognition elements located in a disordered environment, thereby providing a region that maintains its conformational variability upon complex formation [61,62]. The ANP32B LCAR might thus bind to C*c* through specific recognition motifs surrounded by the rest of the disordered LCAR, so making additional contacts with the hemeprotein.

ITC analysis rendered thermodynamic parameters of the interaction between reduced C*c* and different ANP32B constructs. According to the resulting thermograms, C*c* interacts with ANP32B_1-251_ (Fig. 4D, *left panel*) but not with ANP32B_1-161_ (Fig. S2A), in agreement with the aforementioned NMR data. Hence, the LCAR region of ANP32B is clearly the target for C*c* binding.

We fitted the binding isotherms of ANP32B_1-251_ complexed with C*c* to a two different, independent binding sites. The analysis revealed that both C*c* binding sites yielded *K*_D_ values in the micromolar range with significant energetic differences that can be attributed to structural differences at the binding sites (Table 1). We further tested the C*c*:ANP32B complex at higher ionic strength (Fig S2B and S2C). Interestingly, the analysis carried out at 50 mM NaCl yielded *K*_D_ values lower than those obtained in low ionic strength titration experiments, despite the high negative charge of the LCAR (Table S1). This finding could be ascribed to the action of ions shielding the negative charges of the LCAR, which prevents the electrostatic repulsion between the acidic stretches [63]. Therefore, under moderate ionic strength—50 mM NaCl—conditions, the LCAR of ANP32B could be represented by an ensemble of exchanging conformations which, in turn, would facilitate the interaction with C*c*. Moreover, data analysis revealed that the enthalpy value for the higher affinity binding site in the C*c:*ANP32B_1-251_ complex at low ionic strength was substantially more negative than that obtained at 50 mM NaCl (Table 1 and Table S1). This may reflect the contribution of long-range electrostatic interactions during complex formation under low ionic strength, as described for other endogenous C*c* partners and other *c*-type cytochromes [43,48,64,65]. On the other hand, the interaction between ANP32B_1-251_ and C*c* is entropically driven at 50 mM NaCl. This indicates that solvation phenomena are key for the interaction. Such effect could be attributed to intermolecular hydrophobic contacts, but this contrasts with the highly polar nature of both, the LCAR strain and the C*c* surface. Actually, desolvation at charged groups forming salt bridges and the release of ions during complex formation can account for entropy gain in protein-protein interactions as well [66]. Indeed, the weak enthalpy contribution at 50 mM NaCl is compatible with salt bridge formation [66,67]. Experiments carried out at 150 mM NaCl showed that ANP32B interacts with a single molecule of C*c*, being the dissociation constant value larger than that calculated at 50 mM NaCl but similar to that for the binding site with higher affinity at low ionic strength (Table 1 and S1). Interestingly, the entropy contribution was comparable at moderate and high ionic strength, which would suggest a minimal complex rigidification taking place upon binding with minimal entropy penalty (Table S1). Altogether, this data supports a *fuzzy*-type C*c*:ANP32B complex in which the ANP32B LCAR keeps a high degree of disorder (either static or dynamic) as already reported for other—so-called *fuzzy*—complexes involving intrinsically disordered proteins [60]. This is fully consistent with the spread of changes in NMR signals across C*c*, since the ANP32B LCAR samples different areas at C*c* despite maintaining its flexibility.

**Table 1:** Thermodynamic parameters of cytochrome *c*:ANP32B_1-251_ and cytochrome *c*:ANP32B_1-231_ complexes at low ionic strength.

Protein complex	*K*_D_ (μM)	Δ*H* (kcal/mol)	Δ*G* (kcal/mol)	-TΔ*S* (kcal/mol)	*n*
**C*c*:ANP32B**_**1-251**_	9.5	−10.7	−6.8	3.9	0.65
	21.0	29.5	−6.4	−35.9	0.65
**C*c*:ANP32B**_**1-231**_	8.0	−3.6	−6.9	−3.4	1.09

Thermodynamic parameters for the interaction of ANP32B_1-251_ and ANP32B_1-231_ with reduced C*c*. Equilibrium dissociation constant (*K*_D_), association enthalpy (Δ*H*), Gibbs free energy (Δ*G*), entropy (-TΔ*S*) and reaction stoichiometry (*n*) are shown. Relative errors: *K*_D_ 20%, Δ*H* 5%.

The ANP32B LCAR has a highly acidic homogeneous composition, except for the NLS—located at the C-terminal of the acidic tail (Fig. 1A). The ANP32B NLS—placed between residues 239 and 242—is mainly composed by basic amino acids, namely Lys and Arg residues, as other canonical NLS [[68], [69], [70]]. The specific ANP32B NLS is ^239^KRKR^242^; the physico-chemical properties of such a positively-charged NLS remarkably contrast with the spread of negative charges along the rest of the LCAR. We tested whether C*c* explores the surroundings of the NLS using ITC experiments between reduced C*c* and an ANP32B construct lacking the 20-amino acid C-end stretch (ANP32B_1-231_) at low ionic strength (Fig. 4D, *right panel*). Interestingly, the analysis revealed a stoichiometry of 1:1 for C*c* in complex with ANP32B_1-231_ (Table 1); with a *K*_D_ value, as well as with enthalpy and entropy terms very close to the high affinity binding site in the C*c*:ANP32B_1-251_ complex. Consequently, the area surrounding the NLS of ANP32B unequivocally interacts with one molecule of C*c*. Taken together, ITC data suggests that the positively-charged ANP32B NLS (KRKR) might transiently come into contact with any negatively-charged stretch from ANP32B LCAR at low ionic strength, making it difficult for C*c* to bind either NLS or LCAR. This would explain the unfavorable entropic term for the first step of binding for C*c*:ANP32B_1-251_ at low ionic strength (-TΔ*S* = 3.9 kcal/mol, Table 1), due to solvation when the NLS/LCAR is disrupted by C*c*. At moderate ionic strength (50 mM NaCl), the NLS/LCAR interaction may be disrupted, leading to higher affinity for both C*c* binding sites (Table 1 and S1). At high ionic strength (150 mM NaCl), the weaker interaction with C*c* mediated through the NLS is fully disrupted and the stronger LCAR binding site is only partially weakened, yielding a single binding event (Table S1).

### 3.3 Cytochrome *c* competes with histones for ANP32B binding

**Fig. 5:**
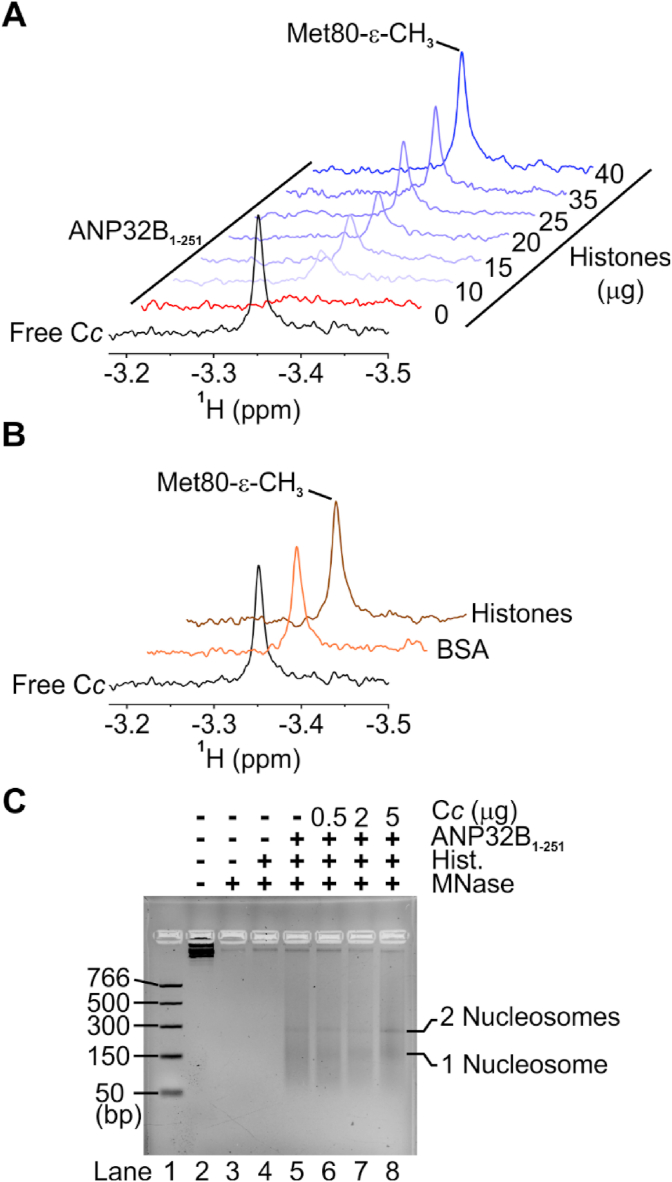
**Cytochrome *c* competes with histones for binding to ANP32B**_**1-251**_**without affecting ANP32B nucleosome assembly activity.** (**A**) Detailed view of the 1D ^1^H NMR spectra monitoring the Met80-ε-CH_3_ signal of reduced C*c* (black trace) in the presence of ANP32B_1-251_ (red trace) and upon adding increasing concentrations of calf thymus histones (blue traces). (**B**) Details of 1D ^1^H NMR spectra of reduced C*c* either free (black) or in the presence of BSA (orange) or histones (brown). (**C**) MNase assay by mixing relaxed plasmid with either histones (lane 4); histones and ANP32B_1-251_ (lane 5) or a mixture of histones, ANP32B_1-251_ and increasing amounts of C*c* (lanes 6-8). Lane 2 and 3 correspond to the free plasmid, untreated and MNase-treated plasmid, respectively. Lane 1 indicates a DNA ladder marker with the size of each band represented on the left. DNA mobilities corresponding to one or two nucleosomes are marked on the right. (For interpretation of the references to color in this figure legend, the reader is referred to the Web version of this article.)

ANP32B is a histone chaperone [12], thus we tested whether C*c* could impair its histone binding activity in competition assays recording 1D ^1^H NMR spectra of reduced C*c* in the presence of ANP32B_1-251_ and increasing histone concentrations. Actually, the addition of ANP32B_1-251_ to a C*c* sample at a 1:0.5 (C*c*:ANP32B) ratio resulted in broadening of the NMR signal from the Met80 ε-methyl proton (Met80-ε-CH_3_) (Fig. 5A). This clearly indicates that ANP32B interacts with the hemeprotein, as observed in complexes of C*c* with other histone chaperones [27,32]. Titration of the C*c*:ANP32B_1-251_ mixture with increasing histone concentrations gradually restored the initial signal (Fig. 5A). Hence, histones can bind to ANP32B_1-251_ and compete with C*c* for ANP32B binding. As a control, the line-width of C*c* Met80-ε-CH_3_ was monitored and found to be insensitive to the addition of either BSA or histones (Fig. 5B).

We then tested if C*c* could inhibit the ability of ANP32B to incorporate nucleosomes into DNA. First, the nucleosome assembly activity of ANP32B_1-251_ was studied using the micrococcal nuclease (MNase) assay, corroborating results reported by Tochio et al. [12] (Fig. 5C, *lane 5*). Surprisingly, increasing concentrations of C*c* had a negligible effect on ANP32B-dependent nucleosome assembly (Fig. 5C, *lanes 6-8*). These results suggest that C*c* competes with histones to bind the LCAR domain of ANP32B, without affecting ANP32B-mediated nucleosome assembly as this activity relies on the LRR domain [12].

### 3.4 ANP32B is a new Protein Phosphatase 2A inhibitor, and cytochrome *c* does regulate such function

**Fig. 6:**
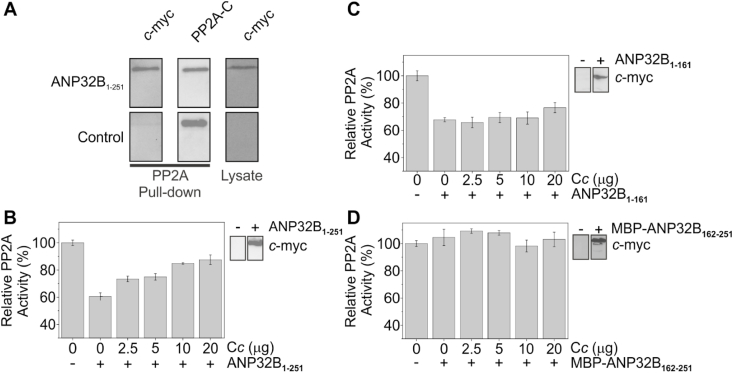
**Cytochrome *c* restores PP2A enzymatic activity by sequestering its inhibitor ANP32B.** (**A**) *Left panel* — Western-Blot against *c*-myc tag, encoded at the C-terminal of ANP32B_1-251_, after performing pull-down assays. *Central panel* — Western-Blot against the catalytic subunit of PP2A (PP2A-C), demonstrating that the endogenous enzyme was captured by means of the Ni-NTA sepharose matrix. *Right panel* — Western-Blot against *c*-myc tag of cell lysates as loading and transfection control. (**B**–**D**) Relative PP2A enzymatic activity in HEK293T cell extracts transfected with either pcDNA 3.1-ANP32B_1-251_-*c*-myc (**B**), pcDNA 3.1-ANP32B_1-161_-*c*-myc (**C**) or pcDNA 3.1-MBP-ANP32B_162-251_-*c*-myc (**D**), upon addition of increasing amounts of recombinant C*c*. Cells transfected with empty pcDNA 3.1 were used as control (*left bar in all panels*). Each activity value corresponds to the average ± S.D. of three independent experiments. Detection by Western-Blot of transfected ANP32B species in cell extracts shown in panels **B**–**D**, by using anti-*c*-myc antibody, are included.

The LRR domain of ANP32B shares an 81% sequence identity and 92% homology with the N-terminal structured domain of ANP32A. Reilly et al. [17] found that the two proteins can actually exchange functions during brain development. As ANP32A has been described as an inhibitor of PP2A [16,71,72], we conjectured that ANP32B could likewise inhibit PP2A activity and that such inhibition could be regulated by C*c*. Therefore, we tested the ability of PP2A to bind ANP32B_1-251_ by pull-down assays using PP2A obtained from HEK293T cell extracts as bait. Strikingly, we detected the interaction between ANP32B_1-251_ and PP2A in total cell extracts (Fig. 6A).

To begin, we tested the ability of ANP32B to hinder PP2A activity and the role that C*c* could play in this activity. Overexpression of ANP32B_1-251_ decreased PP2A activity to *ca*. 60% as compared with control cells. This finding indicates that the histone chaperone ANP32B inhibits PP2A phosphatase activity (Fig. 6B), as does ANP32A. Interestingly, subsequent additions of C*c* led to a *ca*. 90% recovery of PP2A activity (Fig. 6B).

To identify the domain of ANP32B responsible for PP2A inhibition, we performed PP2A enzymatic activity assays in cells overexpressing either truncated ANP32B (ANP32B_1-161_) or the LCAR region only (MBP-ANP32B_162-251_). The LCAR region of ANP32B was cloned at the C-terminal of the MBP to prevent degradation by proteases. As described for the ANP32A LRR domain [16], overexpression of ANP32B_1-161_ inhibited PP2A activity, as did ANP32B_1-251_ (Fig. 6C). However, PP2A inhibition was barely affected by the addition of C*c*. Hence, the LRR domain of ANP32B is responsible for PP2A inhibition. Moreover, ANP32B LCAR over-expression had minimal impact on PP2A activity—then insensitive to the presence of C*c* (Fig. 6D). This strongly suggests that C*c* modulates PP2A inhibition through the LRR of ANP32B by an allosteric effect modulated by the LCAR region.

**Fig. 7:**
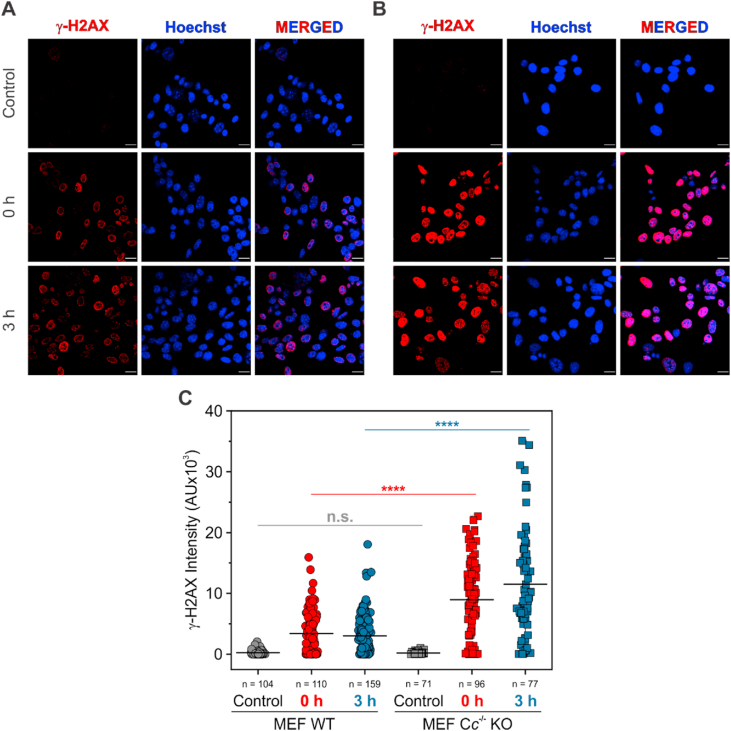
**Cytochrome *c* leads to γ-H2AX dephosphorylation.** Detection of γ-H2AX levels in nuclei of WT (**A**) or C*c*^-/-^ KO (**B**) MEF cells at two recovery times (0 h and 3 h) after treatment with and removal of CPT. Control cells stands for those without CPT treatment. Localization of γ-H2AX (red fluorescence) in cell nuclei is inferred from the merged images in (**A**) and (**B**), in which red fluorescence overlaps with blue Hoechst staining. Scale bars in panels **A** and **B** are 10 μm. (**C**) Scatter plot showing quantification of γ-H2AX signal intensity within the cell nuclei. Black horizontal lines stand for the average values of data collected in each experiment. ****p ≤ 0.0001; n.s., non-significant (one-way ANOVA followed by a Tukey's post hoc test). (For interpretation of the references to color in this figure legend, the reader is referred to the Web version of this article.)

We further delved into the role of C*c* as PP2A modulator by monitoring γ-H2AX levels—a DNA damage hallmark and a PP2A substrate [8]—in MEF WT or C*c*^-/-^ KO cells upon CPT exposure and subsequent recovery periods. Our results showed substantial differences in γ-H2AX intensities between WT and C*c*^-/-^ KO cells at both recovery times (Fig. 7A and B). Quantification of total γ-H2AX fluorescence intensity revealed statistically significant differences in DNA damage levels between the two cell types within the same recovery time (p ≤ 0.0001; Fig. 7C). Strikingly, MEF C*c*^-/-^ KO cells displayed higher γ-H2AX signal intensities after 3-h recovery, in contrast to WT cells, whose γ-H2AX levels slightly decreased within the same timeframe.

**Fig. 8:**
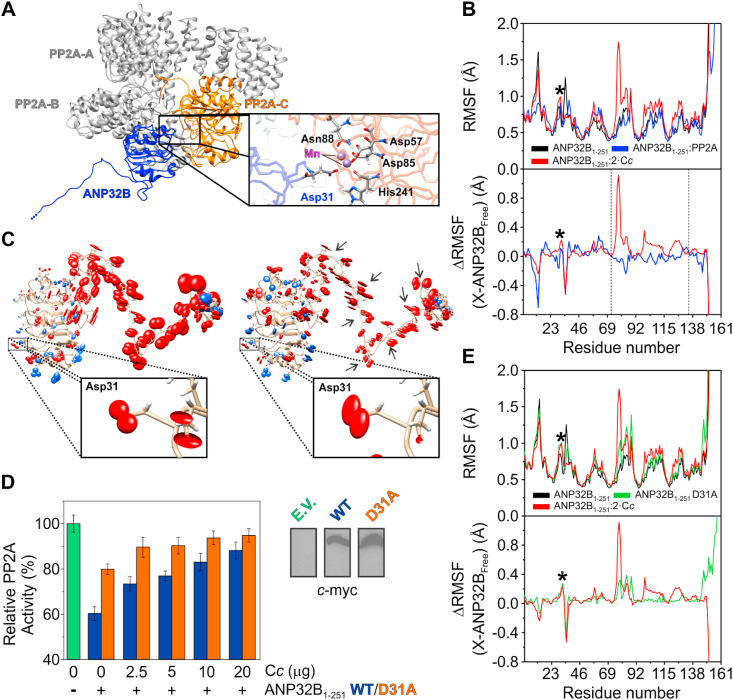
**Long-distance allosteric effects in ANP32B induced by cytochrome *c*.** (**A**) Ribbon representation of ANP32B complexed with PP2A after 10-ns MD. ANP32B is colored in blue, PP2A catalytic subunit (PP2A-C) is represented in orange and PP2A scaffold and regulatory subunits—PP2A-A and PP2A-B respectively—are colored in grey. *Inset* — Detailed view of ANP32B Asp31 side chain—labelled in blue—in close contact with the Mn atoms from PP2A catalytic center. PP2A-C residues in close contact with ANP32B Asp31 residue are labelled in black. (**B**) *Upper panel* — Analysis of the atomic fluctuations (RMSF) of ANP32B LRR domain—comprising residues from 1 to 161—within the context of ANP32B_1-251_ either free (black line), in the presence of 2 molecules of C*c* bound to the LCAR domain (red line) or in complex with PP2A (blue line). *Lower panel* — Subtraction of the RMSF values of ANP32B LRR domain in ANP32B_1-251_ to ANP32B_1-251_:2C*c* (red line) or ANP32B_1-251_:PP2A (blue line) MD data. The dynamic behavior of ANP32B Asp31 residue is marked with an asterisk. (**C**) Representation of the LCAR concerted motions with the LRR domain in ANP32B structure either free (*left panel*) or in the presence of 2 molecules of C*c* placed at LCAR (*right panel*). Red spheroids represent the 3D RMSF values of the oxygen atoms of Glu or Asp sidechains, whereas blue ellipsoids represent 3D RMSF values of nitrogen atoms of Lys and Arg sidechains. A detailed-rotated view of ANP32B Asp31 residue is shown in each panel. Black arrows on *left panel* point out to those residues exhibiting lower atomic fluctuations than in free ANP32B_1-251_ due to C*c* binding. (**D**) Relative PP2A enzymatic activity in HEK293T cell extracts transfected with either empty pcDNA 3.1 (colored in green; E.V. stands for Empty Vector), pcDNA 3.1-ANP32B_1-251_ WT-*c*-myc (colored in blue) or pcDNA 3.1-ANP32B_1-251_ D31A-*c*-myc (colored in orange) upon addition of recombinant C*c* at increasing amounts. As a control, additional WT ANP32B_1-251_ assays were performed in parallel to those for the ANP32B_1-251_ D31A mutant. Each data is the average value ± S.D. of three independent experiments. Detection by Western-Blot of transfected ANP32B species in cell extracts by using anti-*c*-myc antibody are included. (**E**) *Upper panel* — Analysis of the RMSF of the ANP32B LRR domain within the context of free ANP32B_1-251_ WT (black line) and D31A mutant (green line), and in the presence of two molecules of C*c* bound to the LCAR (red line). *Lower panel* — Subtraction of the RMSF values of WT ANP32B LRR domain in ANP32B_1-251_ to ANP32B_1-251_:2C*c* (red line) or ANP32B_1-251_ D31A mutant (green line) MD data. ANP32B Asp31 is marked with an asterisk. (For interpretation of the references to color in this figure legend, the reader is referred to the Web version of this article.)

To unveil into the molecular mechanism underlying C*c*-mediated recovery of PP2A activity we resorted to computational approaches. First, we simulated the ANP32B:PP2A complex. To do this, we performed docking computations between ANP32B_1-161_ and PP2A using Hex Protein Docking [50]. Such computations rely on a rigid-body approach; so artefactual collisions between the flexible LCAR of ANP32B and PP2A could skew the results. Therefore, we used the N-terminal structured domain of ANP32B (ANP32B_1-161_) instead of full-length ANP32B (ANP32B_1-251_) as the ANP32B_1-161_ domain is sufficient to inhibit PP2A (Fig. 6C). The results showed that ANP32B_1-161_ was positioned in the cavity between the regulatory and catalytic subunit of PP2A (i.e. PP2A-B and PP2A-C, respectively; Fig. 8A). Then, we modelled the LCAR domain of ANP32B onto this complex structure and subjected the whole complex to a 10-ns MD simulation to accommodate ANP32B in the PP2A structure and check if ANP32B disassociates from PP2A. Surprisingly, ANP32B_1-251_ kept its original place throughout the trajectory, but a detailed inspection of the PP2A catalytic center revealed that ANP32B Asp31 was pointing towards and in close contact with one of the PP2A Mn atoms (Fig. 8A).

The atomic fluctuations of the LRR domain (residues 1-161) within the full protein (ANP32B_1-251_) changed according to the state of the latter: i) free, ii) with two molecules of C*c* bound at the LCAR—one located at the LCAR C-end next to the NLS and the other at a homogenous, acidic stretch—, and iii) in complex with PP2A (Fig. 8B). Interestingly, fluctuations in ANP32B Asp31 increased in the presence of two molecules of C*c*, as compared with the ANP32B:PP2A complex. This is emphasized by subtracting free ANP32B data to those corresponding to ANP32B:2C*c* or ANP32B:PP2A complexes. Indeed, other regions in the ANP32B_1-161_ domain—such as the stretch comprising residues from 72 to 135—exhibited an alternative behavior when ANP32B was bound to either to two C*c* molecules or to one PP2A molecule. Thus, we hypothesize that the ability of C*c* to block ANP32B-mediated PP2A inhibition relies on two different, synergistic structural effects on the N-terminal region of ANP32B during C*c* binding: i) C*c* induces changes in the dynamics of the ANP32B Asp31 residue, thus altering its correct position in the PP2A catalytic center, and ii) C*c* binding alters the dynamics of the ANP32B LRR domain in the stretch comprising residues 72-135 which, in turn, may hamper ANP32B binding to PP2A.

C*c* is unable to bind to the ANP32B LRR domain, so it impairs PP2A inhibition by means different to inlaying between Asp31 and PP2A. As our experimental data suggests that C*c* could exert long-range allosteric effects on the N-terminal domain of ANP32B upon binding to the LCAR region (see above), we searched for concerted motions involving the LCAR and the LRR. Thus, Principal Component Analyses (PCA) on MD trajectories of ANP32B_1-251_, either free or with two C*c* molecules bound at the LCAR domain were performed. This approach highlighted significant differences in the concerted fluctuations of the sidechains of charged amino acids—e.g. Glu and Asp, mainly located in the LCAR, and Lys and Arg at the LRR (Fig. 8C). Asp31 sidechain motions in particular differed when comparing the two trajectories: LCAR residues in close contact with C*c* molecules exhibited lower atomic fluctuations with respect to free ANP32B_1-251_. As a control, another trajectory was computed for ANP32B_1-161_ (Fig. S3A). Expectedly, an overall damping of motions was evident for all the ionizable sidechains, including Asp31, as compared to the full-construct behavior.

We further tested the role of ANP32B Asp31 in PP2A inhibition assays using HEK293T cell extracts overexpressing an Asp-by-Ala mutant at position 31 (D31A). WT ANP32B_1-251_ was used as a reference (Fig. 8D). The D31A mutant exhibited a lower ability to inhibit PP2A as compared with WT ANP32B_1-251_. In fact, PP2A activity levels were *ca*. 80% of those observed in cells transfected with an empty vector. The addition of increasing amounts of C*c* resulted in faster PP2A activity recovery. Therefore, ANP32B inhibits PP2A enzymatic activity principally by blocking the catalytic center of the enzyme using the Asp31 residue as a cap. An analysis of a MD simulation run on D31A ANP32B_1-251_ revealed that the atomic fluctuations in the N-terminal domain somewhat resembles those observed in ANP32B_1-251_ with two molecules of C*c* bound to the LCAR domain (Fig. 8E). To discard any possible structural alterations due to the Asp-by-Ala mutation, we performed an analysis of the secondary structure in the MD run on WT ANP32B_1-251_ (Figure S3B) and the D31A mutant (Fig. S3C). The point mutation did not significantly alter the overall secondary structure of the ANP32B N-terminal domain, either at long distances or in the adjacent area of the Asp31 residue. Altogether, our data highlights the key role played by ANP32B Asp31 in PP2A inhibition as a single-residue substitution hampers the inhibitory activity of ANP32B without altering its structure.

## 4 Discussion

In this work, we focus on the molecular mechanisms of ANP32B biological functions upon binding to respiratory C*c*, with emphasis on the well-known nucleosome assembly function and novel PP2A inhibitory activity.

Upon DNA damage, endogenous C*c* is promptly translocated into the nucleus where it interacts with endogenous ANP32B, as revealed by colocalization and PLA assays (Fig. 3A, B and 3C). Actually, such nuclear translocation of C*c* occurs within the 6 h timeframe of CPT-induced DNA damage, prior to apoptosome formation and caspase-3 activation (Fig. 2). Despite most ANP32B partners bind at the LRR domain [12,15,16], our NMR and ITC data clearly indicates that C*c* specifically binds to the LCAR domain, which hosts up to two molecules of C*c* in a rather wide conformational space typical of fuzzy ensembles [60]. Moreover, our ITC data on C*c*:ANP32B_1-231_ complex revealed that the hemeprotein specifically recognizes ANP32B NLS and its adjacent area.

**Fig. 9:**
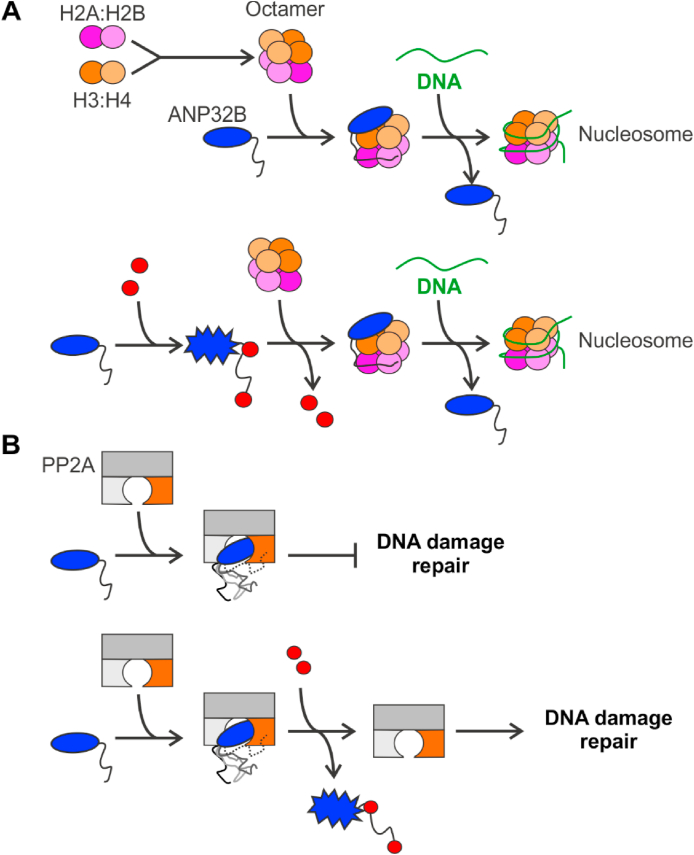
**Functional features of ANP32B upon binding of cytochrome *c* to ANP32B LCAR**. (**A**) *Upper* — ANP32B LRR (blue oval) binds to the histone dimer H3:H4 (orange and pale orange), whereas the ANP32B LCAR domain (black line) binds to the histone dimer H2A:H2B (magenta and pale pink) [12]. Next, ANP32B incorporates the histone octamer into DNA assembling nucleosomes [12,16]. *Lower* — ANP32B LRR undergoes changes in its internal dynamics upon binding of C*c* to the LCAR. H2A:H2B dimer then competes with C*c* for binding to the ANP32B LCAR, thereby releasing C*c* from C*c*:ANP32B ensembles and facilitating the assembly of nucleosomes. (**B**) *Upper* — ANP32B LRR binds to PP2A, whose scaffold subunit is represented in dark grey, the regulatory domain, in light grey; and the catalytic domain, in orange. ANP32B LCAR remains flexible in solution and, eventually, contacts PP2A, thus enhancing the PP2A inhibitory activity of ANP32B_1-251_. PP2A inhibition prevents the activation of DNA damage repair mechanisms, as the histone variant γH2AX and RPA remain phosphorylated. *Lower* — C*c* binding to ANP32B LCAR induces long-distance allosteric effects in the ANP32B LRR domain which, in turn, releases ANP32B from the ANP32B:PP2A ensemble. Free PP2A dephosphorylates γH2AX and RPA, a step necessary to repair damaged DNA [8,[35], [36], [37]]. (For interpretation of the references to color in this figure legend, the reader is referred to the Web version of this article.)

ANP32B is a well-known histone chaperone responsible for assembling nucleosomes around promoters of specific genes, being guided by KLF5 [13]. To fulfil this function, ANP32B binds histone dimers H3–H4 and H2A-H2B through the LRR and LCAR domains, respectively [12]. C*c* competes with histones for ANP32B binding. Nevertheless, C*c* has a negligible effect on nucleosome assembly as the histone chaperone activity of ANP32B relies exclusively on the LRR domain, even though the LCAR domain assists binding of ANP32B to nucleosomes through H2A-H2B histone dimers [12]. Therefore, the nucleosome assembly activity of ANP32B—unlike that of SET/TAF-Iβ [27]—is insensitive to the presence of the hemeprotein (Fig. 9A).

Remarkably, we found that ANP32B is a novel inhibitor of PP2A, alongside the well-established PP2A inhibitors ANP32A and SET/TAF-Iβ [16,34,71,73,74]. PAL31—an ANP32B rat homolog—has been reported to be unable to inhibit PP2A activity [75], however the authors carried out the experimental assays using commercial PP2A devoid of the PP2A-B regulatory subunit. In this work, like in the studies characterizing ANP32A and SET/TAF-Iβ as PP2A inhibitors [16,34,71], heterotrimeric PP2A was obtained directly from cell extracts. Our assays with overexpressed ANP32B LRR or LCAR showed that the structured domain is necessary and sufficient to inhibit PP2A activity, as it is in ANP32A [16]. Differences in the inhibition levels observed with ANP32B_1-251_ and ANP32B_1-161_ suggest that the LCAR may enhance PP2A binding by following two different, non-excluding mechanisms: i) additional contacts between the histone chaperone LCAR domain and PP2A; or ii) changes in the dynamics of the Asp31 residue. Thus, ANP32B may be referred to as I_3_PP2A, alongside I_1_PP2A and I_2_PP2A—the alternative names for ANP32A and SET/TAF-Iβ, respectively.

The novel function of ANP32B is silenced by C*c*, which activates PP2A by liberating the enzyme from its novel inhibitor. In fact, the addition of C*c* at increasing concentrations gradually makes PP2A recover activity. The C*c* dose-response effect was only observed in the assays overexpressing ANP32B_1-251_ and was clearly absent in assays with the ANP32B_1-161_ construct. Such limited influence of C*c* is in agreement with the fact that C*c* interacts with ANP32B exclusively through the LCAR domain, as shown by NMR and ITC experiments. We further corroborated the differential C*c*-dependent modulation of PP2A enzymatic activity in MEF WT and C*c*^-/-^ KO cells by monitoring the fluorescence level of γ-H2AX, which is a PP2A substrate and a DNA damage hallmark. Our results demonstrate that cells lacking C*c* undergo higher DNA damage levels than WT cells—as inferred from higher γ-H2AX fluorescence intensities—especially after 3-h recovery. Altogether, these findings suggest that deficient PP2A-mediated γ-H2AX dephosphorylation in C*c*^-/-^ KO cells might signal the DNA damage site continuously over time, thus preventing the action of the DNA repair machinery.

A detailed analysis of MD calculations revealed that ANP32B Asp31 faces the PP2A catalytic center, blocking the access of PP2A substrates. When two molecules of C*c* bind to the acidic domain, Asp31 side chain motions increase. Additionally, the stretch comprising residues 72-135 exhibits enhanced dynamics with respect to ANP32B, either free or in complex with PP2A. These alterations could act synergistically to release ANP32B from PP2A, yielding a recovery of PP2A enzymatic activity (Fig. 9B). Further assays with PP2A and the ANP32B D31A mutant corroborated the essential role of Asp31 in PP2A inhibition, as the inhibitory activity of the mutant is hampered but quickly recovered upon C*c* binding. Interestingly, our computational data suggests that the ANP32B_1-251_ D31A mutant imitates the effect of C*c* on the N-terminal domain dynamics without altering the overall secondary structure of this domain.

A notable finding from this work is the ability of LCARs to allosterically control the behavior of structured domains within ANP32 proteins. In this way, the unstructured LCAR becomes a long-range molecular sensor capable of modulating some ANP32B functions. According to our data, information can be transmitted across the LCAR and the surface of the LRR domain via a domino effect involving ionic sidechains (Fig. S4A). In this sense, the ANP32B underarm in the structured domain displays some basic residues—i.e. Arg20, Lys65, Lys67 and Lys116—that may couple C-terminal LCAR perturbations to the motions of the N-terminal LRR domain (Fig. S4B). Such a role of Intrinsically Disordered Regions (IDRs) has also been observed in other IDR-containing proteins, namely Ets-1 [76], Sic1 [77] or p27 [78].

Taking everything into account we put forward a model in which C*c* interacts with ANP32B in the nucleus via its unstructured C-terminal LCAR domain, altering the dynamics of the N-terminal structured LRR domain, which could make PP2A shift out of ANP32B. This proposed nuclear function of C*c* reinforces the idea that the hemeprotein plays a regulatory role in the cellular response to DNA damage [79]. We recently proposed that nuclear C*c* alters chromatin dynamics through its interaction with histone chaperones, thereby facilitating DNA repair by maintaining the DNA naked [27,32]. This work expands on this hypothesis as PP2A is a key element in the regulation of the DDR. In fact, PP2A dephosphorylates proteins involved in the DDR, including the phosphorylated histone variant γH2AX [8], Replication Protein A (RPA) [36] and the transducer and effector kinases of the signaling cascade [35,37]. Such modifications are essential for repair machinery activation in response to DNA damage (Fig. 9B). Hence, we hypothesize that the presence of histone chaperones—such as ANP32B—in damaged foci inhibits PP2A activity, thus blocking DNA repair until C*c* reaches the cell nucleus. On the onset of DDR, C*c* is translocated into the cell nucleus [27,28,30,80, this work], where it binds to LCAR-containing histone chaperones—namely SET/TAF-Iβ (NRP1 in plants) [[24], [25], [26]] and ANP32B [this work]—to form a complex network controlling nucleosome (dis)assembly and PP2A-mediated dephosphorylation. Regarding PP2A activity, C*c* binding to ANP32B might dissociate the ANP32B:PP2A complex, with the subsequent increase in the fraction of active PP2A. Our data also indicates that such a complex dissociation upon DNA damage facilitates dephosphorylation of γ-H2AX—either by directly increasing PP2A-dependent dephosphorylation rate or by decreasing the activity of PP2A-regulated ATM and DNA-PK kinases [8,[35], [36], [37]]—in WT MEF cells compared with those lacking C*c*. Recent findings show that C*c* binding to SET/TAF-Iβ hinders the histone chaperone activity of SET/TAF-Iβ [27,79]. The DNA repair machinery can thus access naked DNA at the damaged site while the PP2A activity would also tune the phosphorylation rates of other proteins involved in the DDR signaling [5,8,[35], [36], [37],[81], [82], [83]].

In summary, we propose that respiratory C*c* behaves as a moonlighting protein by playing a dual regulatory role in cell fate decision. First, as a *protector* in the nucleus at the dawn of DDR by altering chromatin dynamics while recovering PP2A activity to facilitate DNA repair and let the cell live. Second, as a *killer* if DNA damage persists or is exacerbated, being massively liberated into the cytoplasm to lead the cell to irretrievable death.
