# Supplementary material for: Inhibition of the PP2A activity by the histone chaperone ANP32B is long-range allosterically regulated by respiratory cytochrome c
Source: Redox Biol. 2021 Apr 18;43:101967. doi: 10.1016/j.redox.2021.101967 (PMC8082267; doi:10.1016/j.redox.2021.101967)
Supplement: Multimedia component 1 [file mmc1.docx]

**Supplementary Figures and Tables**


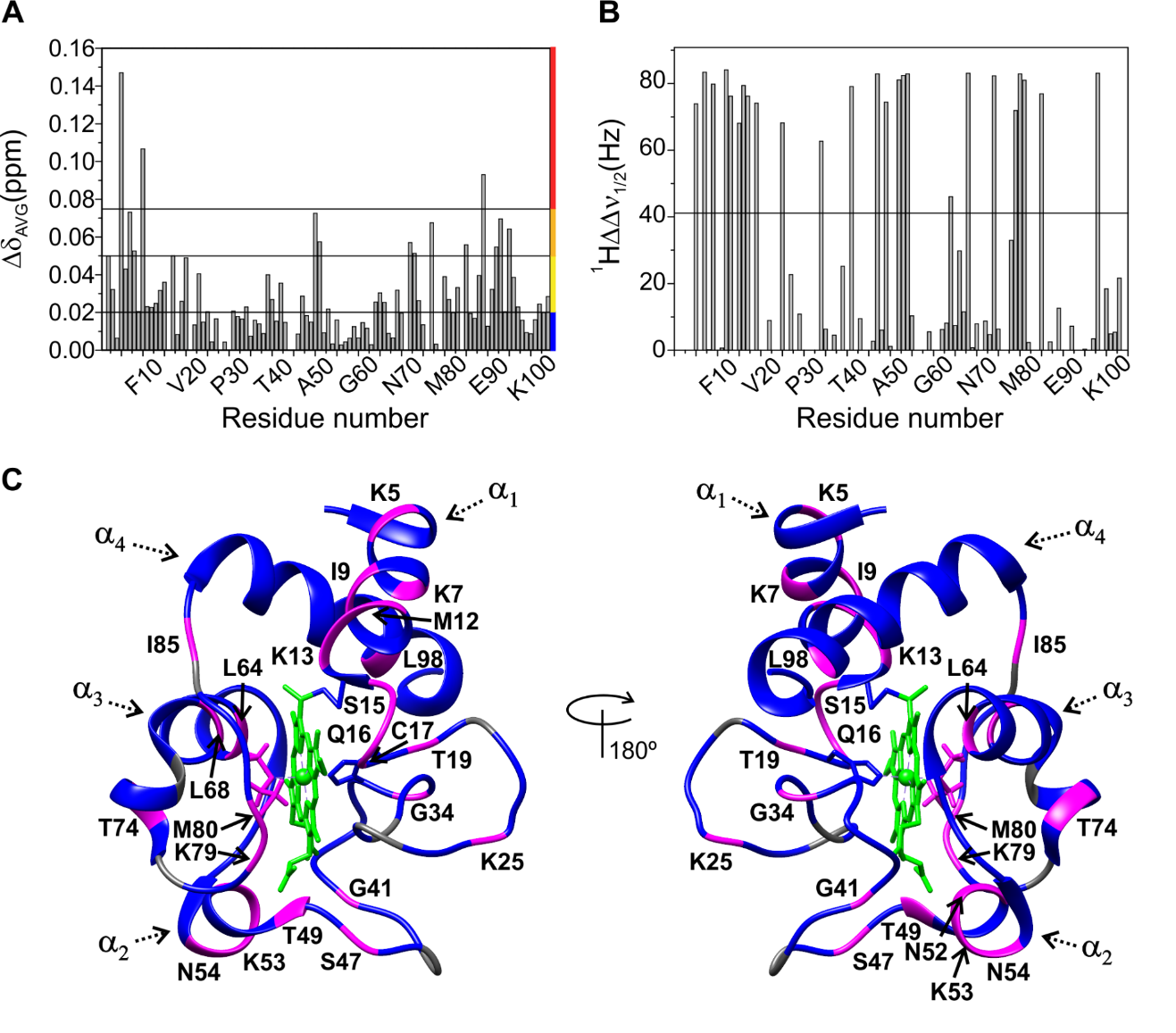


**Figure S1. Cytochrome *c* amide signals show specific chemical-shift perturbations and broadening upon binding to ANP32B_1-251_.** (**A**) Plot of averaged chemical-shift perturbations (Δδ_AVG_) for ^15^N-labeled C*c* titrated with WT ANP32B_1-251_ at a 1:1 molar ratio as a function of residue number. The colored bars represent the Δδ_AVG_ categories as follows: insigniﬁcant <0.025 ppm (blue), small 0.025–0.050 ppm (yellow), medium 0.050–0.075 ppm (orange) and large >0.075 ppm (red). (**B**) Profile of amide line-width broadening (^1^HΔΔν_1/2_) of ^15^N-labeled C*c* titrated with ANP32B_1-251_ at a 1:1 molar ratio as a function of residue number. The threshold, 40.2 Hz, was set for those residues with a broadening intensity higher than the average plus two standard deviations. (**C**) Ribbon representation of C*c* highlighting those amide signals with significant broadening upon binding to ANP32B_1-251_. Residues showing linewidth broadening larger than the average plus two standard deviations are colored in magenta. Prolines and unassigned residues are in grey, and the heme group is in green. C*c* molecule is rotated 180° around vertical axes in each view. The four alpha-helices of C*c*—named from α_1_ to α_4_—are indicated by dashed arrows. C*c* PDB ID: 1J3S [47].


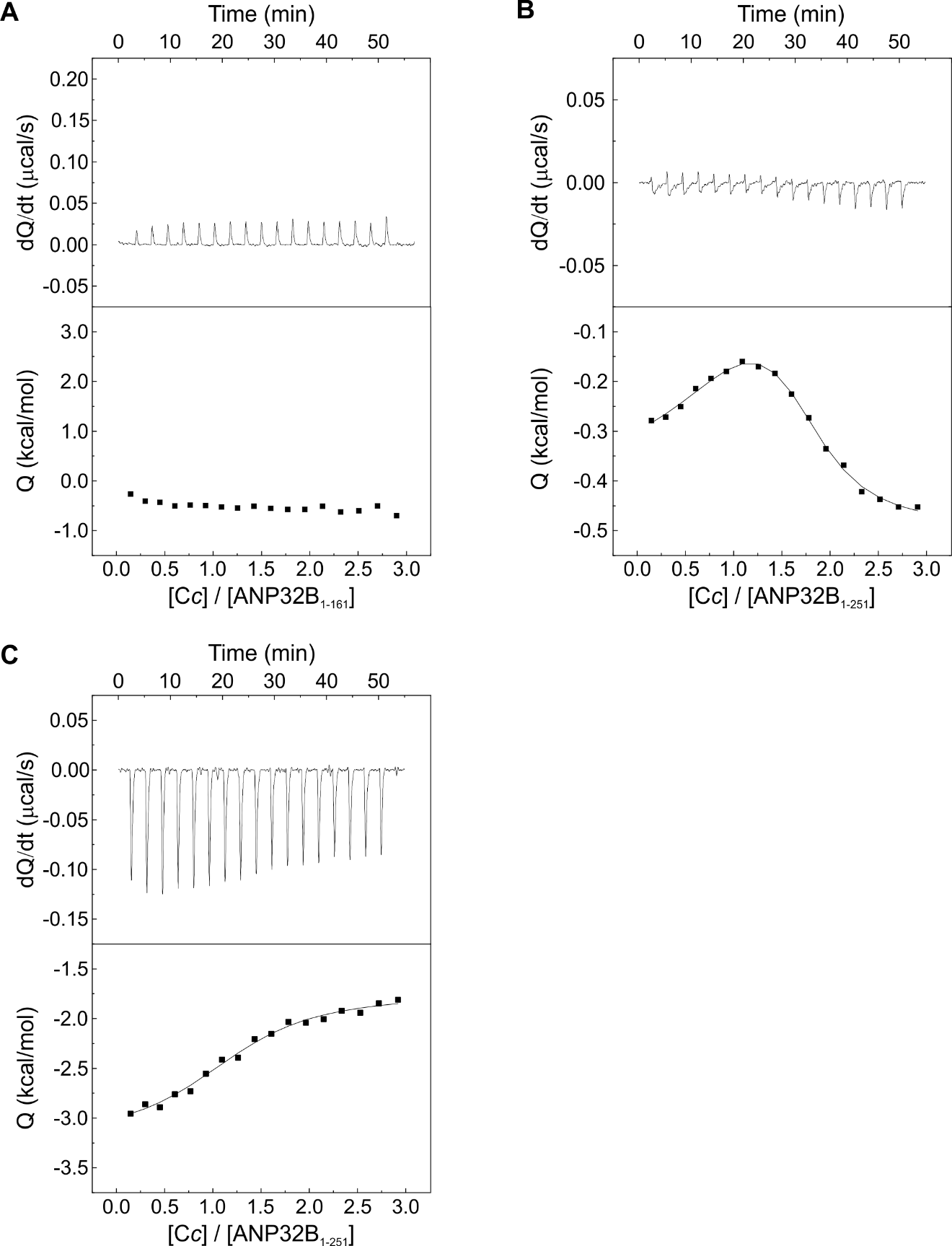


**Figure S2. ITC binding experiments with cytochrome *c* and ANP32B_1-161_ or ANP32B_1-251_ at high ionic strength.** (**A**) ITC titration data between C*c* and ANP32B_1-161_ does not display any hint of a complex formation. (**B**-**C**) Binding experiments were performed using reduced C*c* and ANP32B_1-251_ in 20 mM sodium phosphate buffer pH 7.4 with 50 mM NaCl (**B**) or 10 mM sodium phosphate pH 7.4 with 150 mM NaCl (**C**). Thermograms and binding isotherms are shown in the *upper* and *lower* panels, respectively.


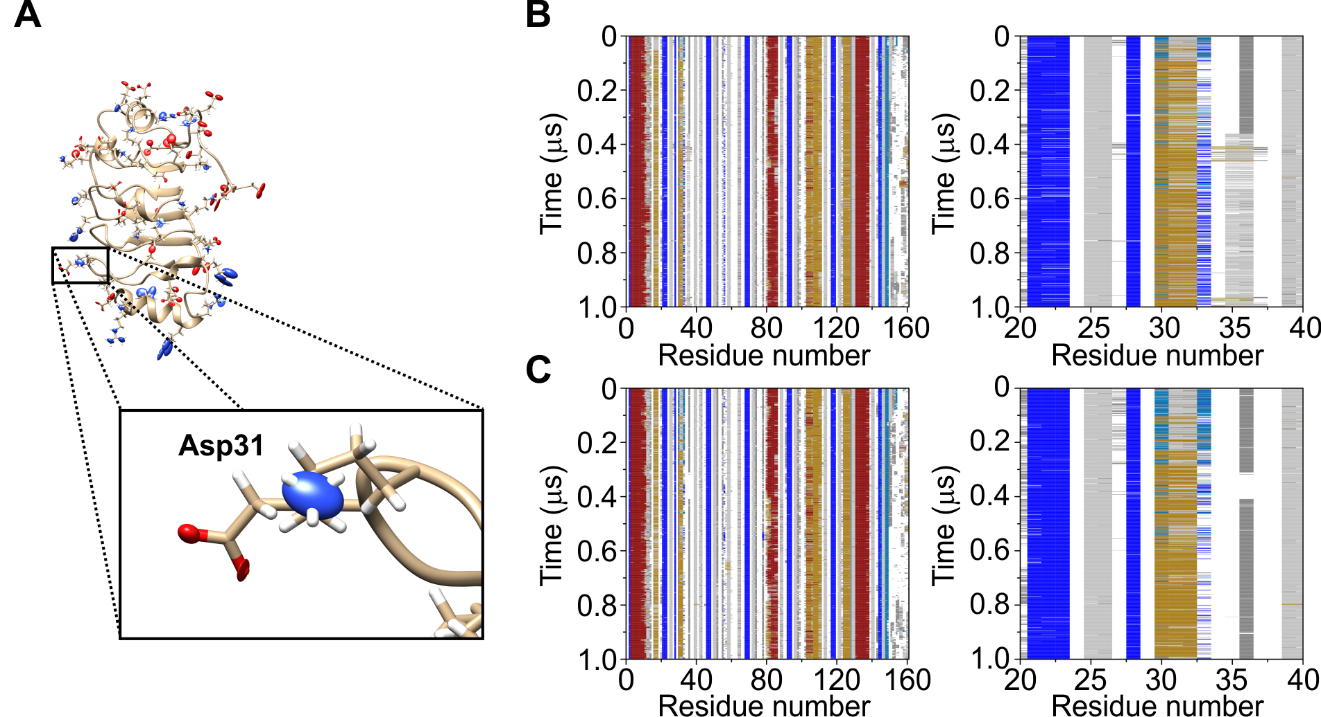


**Figure S3. Controls of Molecular Dynamics data herein presented.** (**A**) Representation of the MD-inferred concerted movements in the ANP32B_1-161_ construct. Red spheroids represent the 3D RMSF values of the oxygen atoms of Glu or Asp sidechains, whereas blue spheroids represent 3D RMSF values of nitrogen atoms of Lys and Arg sidechains. A detailed view of ANP32B Asp31 residue is shown. (**B**-**C**) Representation of the evolution in secondary structure of the LRR domain in MDs run on ANP32B_1-251_ (**B**) and ANP32B_1-251_ D31A mutant (**C**). Parallel β-sheets are colored in blue; anti-parallel β-sheets, in dark blue; 3_10_ helices, in khaki; α-helices, in red; turns, in light grey; bends in dark grey; and those elements exhibiting no secondary structure, in white. A detailed view of the Asp31 residue adjacent area is shown in right panels **B** and **C**.


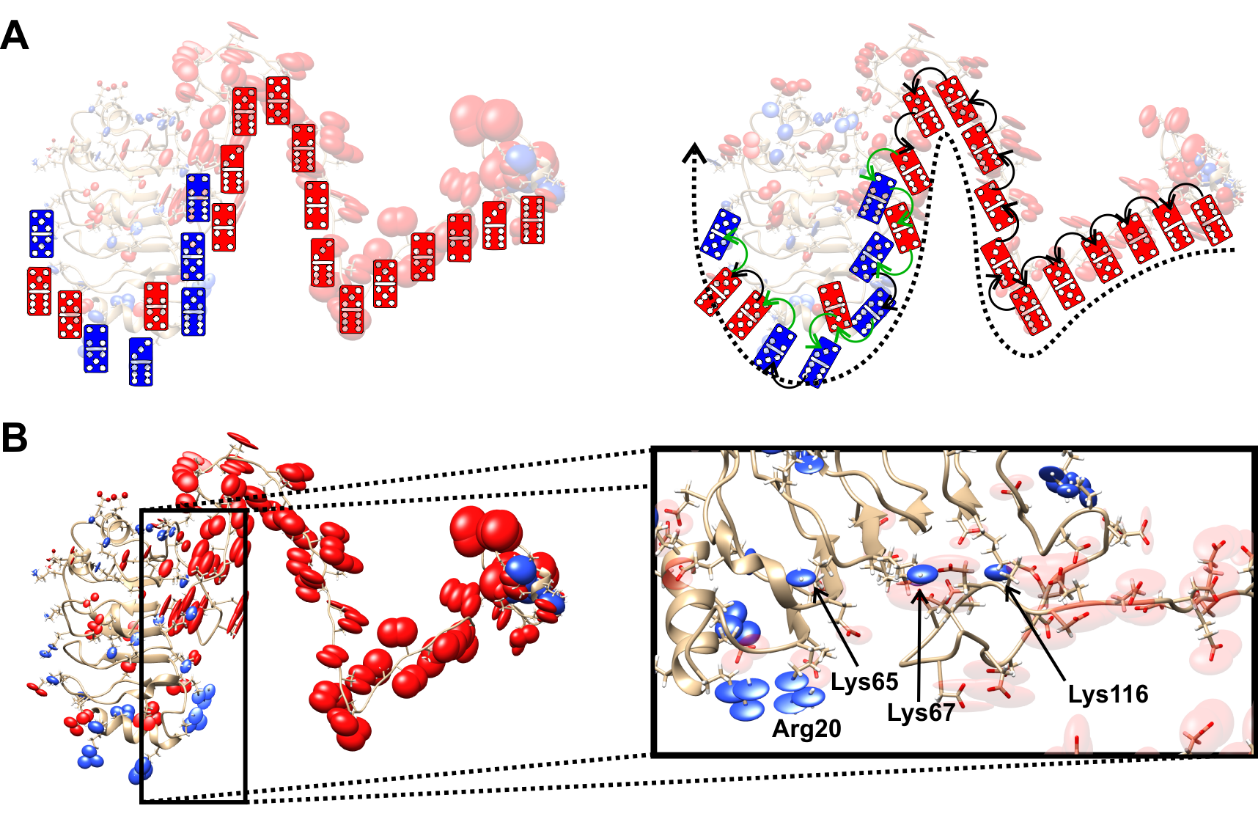


**Figure S4. ANP32B low complexity acidic region acts as a sensor, transmitting information to the structured domain through a side-chain domino effect.** (**A**) *Left* — ANP32B LCAR contains a high number of negatively-charged amino acids (red dominoes), whereas ANP32B LRR exhibit both acidic and basic amino acids (blue dominoes). *Right* — Upon a perturbation onto the LCAR domain, such as the binding of two C*c* molecules, the information is transmitted to the N-terminal structured domain through both repulsion and attraction of ionic sidechains (black and green arrows, respectively). (**B**) Detailed view of the positively charged amino acids placed at ANP32B armpit which allows the information to pass from the LCAR to the LRR domain. For panels **A** and **B**, red spheroids stand for the 3D RMSF value calculated in ANP32B_1-251_ MDs of the oxygen atoms of Glu or Asp sidechains, whereas blue spheroids stand for 3D RMSF values for nitrogen atoms of Lys and Arg sidechains.

**Table S1. Thermodynamic parameters of the cytochrome *c*:ANP32B_1-251_ complex at moderate and high ionic strength**

| **Protein complex** | **[NaCl]**  **(mM)** | ***K*_D_**  **(μM)** | **Δ*H***  **(kcal/mol)** | **Δ*G***  **(kcal/mol)** | **-TΔ*S***  **(kcal/mol)** | ***n*** |
| --- | --- | --- | --- | --- | --- | --- |
| **C*c*:ANP32B_1-251_** | 50 | 1.1 | 0.1 | -8.0 | -8.1 | 0.86 |
|  |  | 4.4 | 0.6 | -7.3 | -7.9 | 0.86 |
| **C*c*:ANP32B_1-251_** | 150 | 9.1 | -1.5 | -6.8 | -5.3 | 1.20 |

Thermodynamic parameters for the interaction between ANP32B_1-251_ and reduced C*c* at two ionic strength values. Equilibrium dissociation constant (*K*_D_), association enthalpy (Δ*H*), Gibbs free energy (Δ*G*), entropy (-TΔ*S*) and reaction stoichiometry (*n*) are shown. Relative errors: *K*_D_ 20%, Δ*H* 5%.
